# Human local adaptation of the TRPM8 cold receptor along a latitudinal cline

**DOI:** 10.1371/journal.pgen.1007298

**Published:** 2018-05-03

**Authors:** Felix M. Key, Muslihudeen A. Abdul-Aziz, Roger Mundry, Benjamin M. Peter, Aarthi Sekar, Mauro D’Amato, Megan Y. Dennis, Joshua M. Schmidt, Aida M. Andrés

**Affiliations:** 1 Department of Evolutionary Genetics, Max Planck Institute for Evolutionary Anthropology, Leipzig, Germany; 2 Department of Archaeogenetics, Max Planck Institute for the Science of Human History, Jena, Germany; 3 Max Planck Institute for Evolutionary Anthropology, Leipzig, Germany; 4 Department of Human Genetics, University of Chicago, Chicago, Illinois, United States of America; 5 Genome Center, MIND Institute, and Department of Biochemistry & Molecular Medicine, University of California, Davis, California, United States of America; 6 BioDonostia Health Research Institute and IKERBASQUE, Basque Foundation for Science, San Sebastian, Spain; 7 UCL Genetics Institute, Department of Genetics, Evolution and Environment, University College London, London, United Kingdom; National Institute of Genetics, JAPAN

## Abstract

Ambient temperature is a critical environmental factor for all living organisms. It was likely an important selective force as modern humans recently colonized temperate and cold Eurasian environments. Nevertheless, as of yet we have limited evidence of local adaptation to ambient temperature in populations from those environments. To shed light on this question, we exploit the fact that humans are a cosmopolitan species that inhabit territories under a wide range of temperatures. Focusing on cold perception–which is central to thermoregulation and survival in cold environments–we show evidence of recent local adaptation on *TRPM8*. This gene encodes for a cation channel that is, to date, the only temperature receptor known to mediate an endogenous response to moderate cold. The upstream variant rs10166942 shows extreme population differentiation, with frequencies that range from 5% in Nigeria to 88% in Finland (placing this SNP in the 0.02% tail of the F_ST_ empirical distribution). When all populations are jointly analyzed, allele frequencies correlate with latitude and temperature beyond what can be explained by shared ancestry and population substructure. Using a Bayesian approach, we infer that the allele originated and evolved neutrally in Africa, while positive selection raised its frequency to different degrees in Eurasian populations, resulting in allele frequencies that follow a latitudinal cline. We infer strong positive selection, in agreement with ancient DNA showing high frequency of the allele in Europe 3,000 to 8,000 years ago. rs10166942 is important phenotypically because its ancestral allele is protective of migraine. This debilitating disorder varies in prevalence across human populations, with highest prevalence in individuals of European descent–precisely the population with the highest frequency of rs10166942 derived allele. We thus hypothesize that local adaptation on previously neutral standing variation may have contributed to the genetic differences that exist in the prevalence of migraine among human populations today.

## Author summary

Some human populations were likely under strong pressure to adapt biologically to cold climates during their colonization of non-African territories in the last 50,000 years. Such putative adaptations required genetic variation in genes that could mediate adaptive responses to cold. *TRPM8* is potentially one such gene, being the only known receptor for the sensation of moderate cold temperature. We show that a likely regulatory genetic variant nearby *TRPM8* has several signatures of positive selection raising its frequency in Eurasian populations during the last 25,000 years. While the genetic variant was and is rare in Africa, it is now common outside of Africa, with frequencies that strongly correlate with latitude and are highest in northern European populations. Interestingly, this same genetic variant has previously been strongly associated with migraine. This suggests that adaptation to cold has potentially contributed to the variation in migraine prevalence that exists among human groups today.

## Introduction

While human ancestors lived in Africa for millions of years, their successful colonization of colder environments outside of Africa is relatively recent, occurring during the last ~50,000 years. A number of novel genetic adaptations in populations that settled extreme polar environments are documented [1–3]. This includes an allele in the gene *CPT1A*, which encodes a protein involved in the regulation of mitochondrial oxidation of fatty acids, in Northern Siberian populations [1, 2], and several alleles in genes involved in fatty acid metabolism in Greenlanders [3, 4]. These genetic changes likely represent adaptations to the highly specialized diets of these specific populations, which are rich in fatty acids. However, the putative adaptations to temperature and climate are largely unresolved.

Even in non-polar environments, temperatures range substantially across human habitats. For example, average annual temperature is 28°C in Nigeria (home to the Yoruba) and only 6°C in Finland, with differences most pronounced from December to February (29°C in in Nigeria and -4°C in Finland). These temperature differences illustrate the habitat changes experienced by early human groups as they migrated north. Local adaptation has significantly contributed to population differentiation that exists among human populations [5]. So it is reasonable to expect that besides genetic adaptations to selective factors that correlate with climate, such as diet [1–3] and subsistence strategy [6], or pathogens [7] and their load [8], humans may harbor direct genetic adaptations to temperature and other climatic factors [6, 9].

Thermosensation (the sensation of innocuous environmental temperature) is crucial for thermoregulation (the process that maintains core body temperature) and is mediated by warm and cold receptor nerves that innervate the skin. At the molecular level, temperature sensation is due to the activation of transient receptor potential (TRP) ion channels. Among the few TRPs with clear thermoregulatory role (reviewed in [10]), only TRP cation channel subfamily M member 8 (TRPM8) is broadly agreed to play a central role in cold sensation and subsequent physiological thermoregulation [11–17]. *TRPM8* is expressed in pain and temperature-sensitive neurons of the dorsal root ganglia [15], and at lower levels in other tissues such as prostate and liver [10, 18]. From approximately 15°C to 30°C the channel passes a mixed inward cationic current with strength inversely proportional to temperature. Interestingly, it is also activated by natural ligands such as menthol [17, 19] and is responsible for the local cooling sensation of mint-containing products [19]. Proof of its physiological role in thermoregulation is that its deletion diminishes responses to cold [11–13] including behavioral responses to innocuous cool, noxious cold, injury-evoked cold hypersensitivity and cooling-mediated analgesia [20]. In fact, TRPM8 is the only well-stablished cold receptor and, as such, a prime candidate to have mediated putative adaptations to cool and cold environments. Strikingly, it was recently shown that a few substitutions in the TRPM8 transmembrane domain are responsible for the reduced sensitivity to cold of two different hibernating rodents, when compared with non-hibernating species [21]. This further points to *TRPM8* as the most obvious candidate to investigate cold adaptation in humans.

*TRPM8*, located on the short arm of human chromosome 2, harbors genetic diversity with potential functional and phenotypic consequences. Specifically, a single-nucleotide polymorphism (SNP; rs10166942, C/T, chr2:234825093 in hg19) upstream of the gene is predicted to alter transcription factor binding [22] and shows genetic association with phenotypic variation. The SNP is strongly associated with migraine in Europeans, with the ancestral C allele being protective of migraine with and without aura [23–26] with an effect that is among the largest in the genome (e.g. odds ratio = 0.89–0.99, p-value = 1.0 x 10^−23^ in [25]). The precise molecular mechanism for this association remains unknown, although TRPM8 likely plays a role in pain perception at least with noxious cold stimuli and peripheral inflammation (reviewed in [27, 28]), and the channel mediates the analgesic effect of menthol in acute and inflammatory pain [29]. Interestingly, migraine leads to increased pain perception of non-noxious cold temperature [30] and ingestion of cold water can in some cases trigger migraine [31], providing possible links between TRPM8’s mediated cold perception and some aspects of migraine. Of note, rs10166942 has also been recently associated with irritable bowel syndrome (IBS) with constipation in Swedish cohorts (odds ratio = 1.91, p-value = 5.1 x 10^−05^) [22]. This result suggests a possible gastrointestinal function for TRPM8 and rs10166942, although the sample sizes were small, the association has not yet been replicated, and the putative molecular mechanisms remain unknown.

TRPM8’s role in cold perception and thermoregulation, together with its role in temperature adaptation in hibernating rodents, suggest that *TRPM8* has the potential to mediate adaptations to cold ambient temperature in humans. Here, we use a combination of genetic methods to resolve the evolutionary history of *TRPM8* in human populations, and show strong evidence for local adaptation that correlates with latitude and temperature.

## Results

To investigate the recent evolutionary history of *TRPM8*, we focused on the rs10166942 SNP following several lines of evidence that suggest functional relevance. The first one is association with disease, as the ancestral C allele shows strong association with reduced risk of migraine [24] that has been consistently replicated in different populations e.g. [23, 25, 26, 32], although the molecular mechanism responsible for these associations remains unknown. This is most likely due to the restricted tissue expression of the gene and the temperature/ligand-dependent activation of the protein, which severely hamper experimental functional assays (S1 Fig)–as, for example, typical genome-wide experiments are run under basal conditions [33]. It is worth noting that computational predictions suggest rs10166942 alters transcription factor binding [22]. The very specific tissue expression of the gene makes it extremely challenging to test this prediction experimentally, but a regulatory function fits well the location of the SNP, which sits ~1 kb upstream of *TRPM8*. We note that no neighboring SNP in high linkage disequilibrium (LD) shows stronger evidence of association with migraine [24] or functionality (S2 Fig) than rs10166942. Thus, rs10166942 remains as the most likely functional variant in this genomic region and we chose it as our target variant–with the understanding that we cannot discard the possibility that it tags another functional variant in this locus which would, however, share its genetic signatures.

### Latitude and *TRPM8*-rs10166942

**Fig 1:**
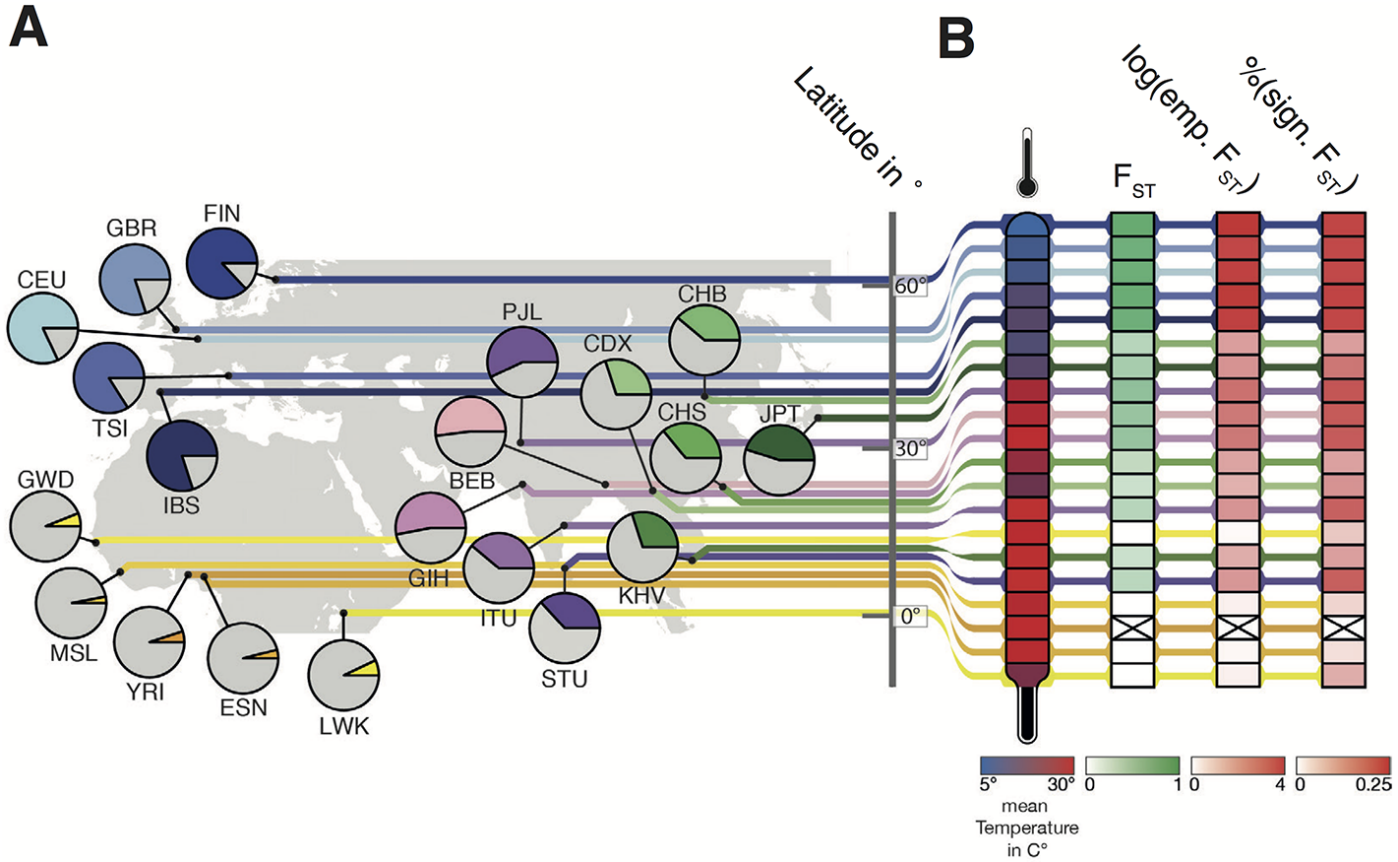
Overview of the populations used and their allele frequencies for rs10166942, average temperature, and F_ST_ signatures. **(A)** Geographic location of the 1KGP populations used, with the derived allele frequency of the rs10166942 allele in pie charts (T allele in color according to population), and their latitude. **(B)** In columns, annual mean temperature at the geographic location of each population, the level of F_ST_-based population differentiation with YRI, the log_10_ empirical P-value of this F_ST_ value, and the proportion of SNPs in the 65 kb target region with an empirical P-value lower than 0.05.

The rs10166942 variant shows interesting patterns of allele frequencies in the 1000 Genomes populations (hereafter 1KGP) [34] (Fig 1A, Table 1). The derived T allele is not introgressed (not in identified introgressed segments [35] and absent in the sequenced Neandertals and Denisovan genomes [36–38]), and levels of linked variation indicate that it originated in Africa (S3 and S4 Figs, S1 Table). Still, its frequency today is just 5% in the equatorial YRI, but it reaches intermediate frequencies in Asia and up to 88% frequency in the northern European Finnish (Fig 1A, Table 1). Frequencies of the rs10166942 T allele in South Asia are on average 0.48, closer to those in East Asia (0.36) than in Europe (0.83), in contrast with the patterns of shared ancestry–genome-wide South Asian populations are closer to Europeans than to East Asians (S5 Fig) [34]. Together, allele frequencies paint a seemingly latitudinal cline of allele frequencies (Fig 1A, Table 1).

**Table 1:** Overview of populations and signatures of natural selection. Geographic coordinates (in degrees), mean annual temperature (in degrees Celsius), and the frequency and signatures of selection for the rs10166942 T allele (empirical P-value), per population, ordered by latitude. DAF: derived allele frequency. Continents: (EUR) Europe, (EAS) East Asia, (SAS) South Asia, (AFR) Africa.

Population	Continent	Latitude	Longitude	Temperature	DAF	F_ST_	F_ST_ P-value	XP-EHH P value	iHS P-value
FIN	EUR	60,25N	24,75E	5.7	0.87	0.805	0.0002	0.205	0.166
GBR	EUR	54,75N	1,25W	10.0	0.80	0.724	0.0006	0.287	0.304
CEU	EUR	50,75N	4,25E	10.7	0.82	0.751	0.0004	0.228	0.36
TSI	EUR	43,25N	11,25E	14.2	0.84	0.778	0.0002	0.26	0.622
IBS	EUR	40,25N	3,25W	14.9	0.80	0.733	0.0004	0.291	0.656
CHB	EAS	39,75N	116,25E	13.4	0.39	0.279	0.0550	0.939	0.219
JPT	EAS	35,25N	139,25E	14.8	0.45	0.349	0.0356	0.947	0.593
PJL	SAS	31,25N	74,25E	25.3	0.57	0.472	0.0066	0.651	0.869
BEB	SAS	23,25N	90,25E	26.1	0.52	0.428	0.0102	0.605	0.8
GIH	SAS	23,25N	72,75E	27.7	0.53	0.437	0.0101	0.587	0.821
CHS	EAS	22,25N	114,25E	23.4	0.36	0.254	0.0666	0.927	0.161
CDX	EAS	22,25N	100,25E	19.2	0.30	0.184	0.1051	0.895	0.926
ITU	SAS	16,75N	80,75E	28.6	0.39	0.278	0.0367	0.804	0.952
GWD	AFR	13,25N	16,25W	27.2	0.06	-0.007	0.8610	NA^b^	0.39
KHV	EAS	10,25N	106,25E	28.2	0.30	0.193	0.0953	0.938	0.69
ESN	AFR	6,75N	6,25E	27.0	0.04	-0.007	0.8046	NA^b^	NA^c^
STU	SAS	9,25N	80,25E	28.5	0.37	0.262	0.0411	0.866	0.626
MSL	AFR	7,75N	11,25W	26.6	0.03	-0.005	0.6836	NA^b^	NA^c^
YRI	AFR	7,25N	3,75E	27.6	0.05	NA^a^	NA^a^	NA^a^^,^ ^b^	0.699
LWK	AFR	0,75N	34,75E	20.5	0.07	-0.002	0.6912	NA^b^	0.901

^a^ Not calculated because YRI was used as background population.

^b^ XP-EHH not calculated within Africa.

^c^ Allele frequency did not meet criteria (see Methods).

**Fig 2:**
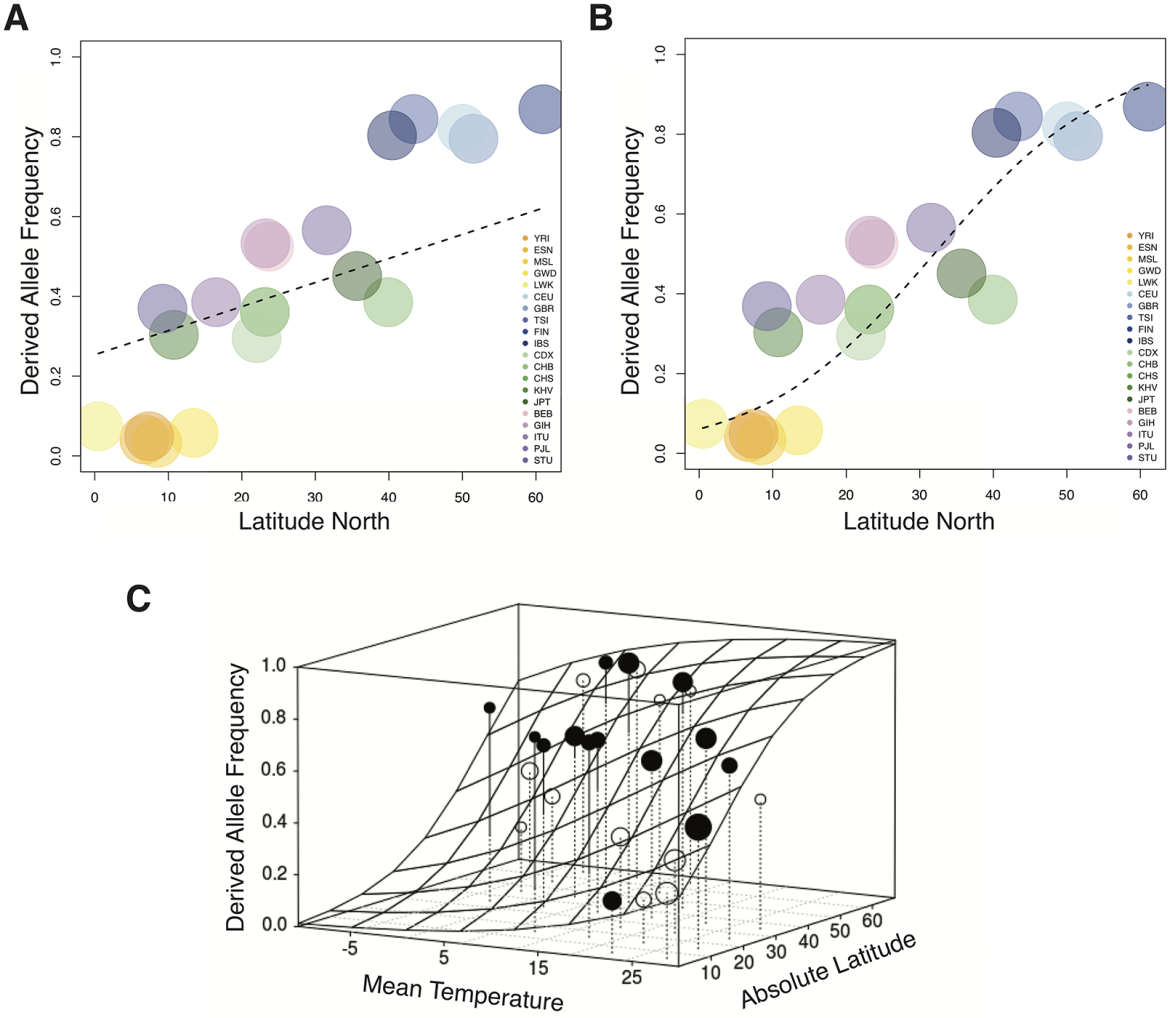
Correlation between latitude and derived allele frequency. Correlation of the frequency of the rs10166942 T allele with latitude. The fitted function (dashed line) results for the 1KGP data from **(A)** the PGLS and **(B)** GLMM analysis. **(C)** Results of the best model in the GLMM analysis of the SGDP dataset. The fitted response is shown as gridded surface, and the dots represent the average frequency of the rs10166942 T allele per cell of the gridded surface. Points above the surface are filled, points below are open. The volume of the points corresponds to the number of populations per cell.

We tested this hypothesis using linear models and, because of the thermoregulatory role of *TRPM8*, included temperature as a covariate. We tested, using a Phylogenetic Generalized Least Square (PGLS) [39] analysis, to what extent shared ancestry, latitude and annual average temperature predict the observed allele frequency in each population. PGLS is an extension of the general linear model that analyzes the impact of one or several predictor variables (here, latitude and temperature) on a single response variable (allele frequencies) while controlling for the phylogenetic signal (the correlation in allele frequencies across populations due to shared ancestry) [40]. We first performed a model comparison between a null model (only ancestry information) and a full model (which includes latitude and temperature as predictor variables). The full model explains the data significantly better than the null model (χ^2^ = 13.04, df = 2, P-value = 0.001). When we then assessed the influence of each predictor with multi model inference, the null model again receives weak support (Table 2). The highest support is for the model with latitude, followed closely by the model with latitude and temperature; together, they make up the 95% best model confidence set (Table 2), placing latitude alone or combined with temperature as a better predictor of rs10166942 T allele frequency than shared ancestry. The correlation between allele frequency and latitude in this model is evident in Fig 2A. We also used a Generalized Linear Mixed Model (GLMM), which uses one-dimensional ancestry information but allows non-linear fits to the data and can use genotype data. This confirmed the significant latitude correlation, with and without temperature, in 1KGP data (Fig 2B; Table 2). In addition, we confirmed this result using 110 populations of the Simons Genome Diversity Project (SGDP) dataset (Supplemental Data 1) [41], which provide a much denser worldwide population sample (Fig 2C, S6 Fig, Table 2). Further, the significant correlation remains when only Eurasian populations are analyzed (in the SGDP dataset, where the number of populations allows this analysis), showing that the inference is not driven by the low frequency of the T allele in African populations (Table 2).

**Table 2:** PGLS and GLMM analysis. All models considered, ordered by their fit (Model rank). Three measures of model support are shown: AIC, delta AIC, and Akaike weight. The cumulative probability are shown together with the resulting confidence set (models that together provide just over 0.95 cumulative probability; indicated by ‘yes’). Results are shown for the 1KGP in PGLS and GLMM analyses, the SGDP in a GLMM analysis, and the SGDP using only the Eurasian populations in a GLMM analysis.

	Models*	Model Rank	AIC	delta AIC	weight AIC	cumulative Pr.	confid. Set	k^#^
**1KGP** **PGLS**	Null+Lat.	1	-49.43	0	0.504	0.504	yes	5
	Null+Temp.+Lat.	2	-49.186	0.244	0.446	0.95	yes	6
	Null+Temp.	3	-44.147	5.283	0.036	0.986	no	5
	Null	4	-42.255	7.175	0.014	1	no	4
**1KGP** **GLMM**	Null+Temp.+Lat.	1	1929.2	0	0.510	0.510	yes	6
	Null+Lat.	2	1929.3	0.09	0.488	0.997	yes	5
	Null+Temp.	3	1939.8	10.633	0.003	1	no	5
	Null	4	1946.2	16.964	0	1	no	4
**SGDP** **GLMM**	Null+Temp.+Lat.	1	435.206	0	0.943	0.943	yes	6
	Null+Lat.	2	440.841	5.635	0.056	1	yes	5
	Null+Temp.	3	451.699	16.494	0	1	no	5
	Null	4	452.458	17.252	0	1	no	4
**SGDP** **Eurasia** **GLMM**	Null+Lat.	1	301.301	0	0.582	0.581	yes	5
	Null+Temp.+Lat.	2	302.35	1.05	0.344	0.926	yes	6
	Null+Temp.	3	305.874	4.574	0.059	0.985	yes	5
	Null	4	308.611	7.311	0.015	1	no	4

*Models: Lat…Latitude; Temp…Temperature

^#^ k: number of estimated parameters

Latitude is thus a strong predictor of genotype–that is, of the presence and frequency of the rs10166942 T allele in a given population. Temperature is a weaker predictor, perhaps because it is less stable over time. Available genomic data from prehistoric Eurasians (ages 3,000 to 8,500 years old [42, 43]) show no significant support for any predictor (Materials and methods; S7 Fig), although the low number and restricted geographic origin of these ancient samples markedly hamper the analysis. In any case, ancient DNA suggests that the derived rs10166942 T allele was already at high frequencies in pre-historic European groups that include Hunter-Gatherers (frequency 81%), Farmers (77%), Steppe pastoralists (71%) and possibly Paleo-Eskimos from Greenland (the available genome is T homozygote) [43].

### Signatures of positive selection at *TRPM8*-rs10166942

The observation that rs10166942 frequencies are better explained by latitude than population history, with extremely high frequencies of the T allele in Northern Europe, raises the possibility that adaptation to north Eurasian environments resulted in increased frequency of this *TRPM8* allele. We first explored signatures of local positive selection using F_ST_, a measure of population differentiation to the equatorial YRI population. rs10166942 is among the most strongly differentiated SNPs genome-wide between YRI and not only all European populations (GBR, FIN, IBS, TSI, CEU; empirical P-values = 0.0002–0.0006), but also all South Asian (STU, ITU, GIH, BEB, PJL; P-values = 0.041–0.007), and one East Asian (JPT; P-value = 0.0356) population (Fig 1B, Table 1). The high F_ST_ signature extends for ~65 kb in the upstream half of *TRPM8* and, due to LD, some SNPs show comparable signatures, but only rs10166942 has been associated with a phenotype (S8 Fig). F_ST_ sharply declines beyond the 65kb upstream portion of *TRPM8*, probably due to recombination (S8 and S9 Figs). Although non-African populations show relatively high LD in the locus (S9 Fig), LD-based statistics show weak evidence of population-specific (XP-EHH [44]) or incomplete (iHS [45]) selective sweeps on a new advantageous mutation at rs10166942 and nearby SNPs (Table 1, S8 Fig).

### Evolutionary history of TRPM8-rs10166942

**Fig 3:**
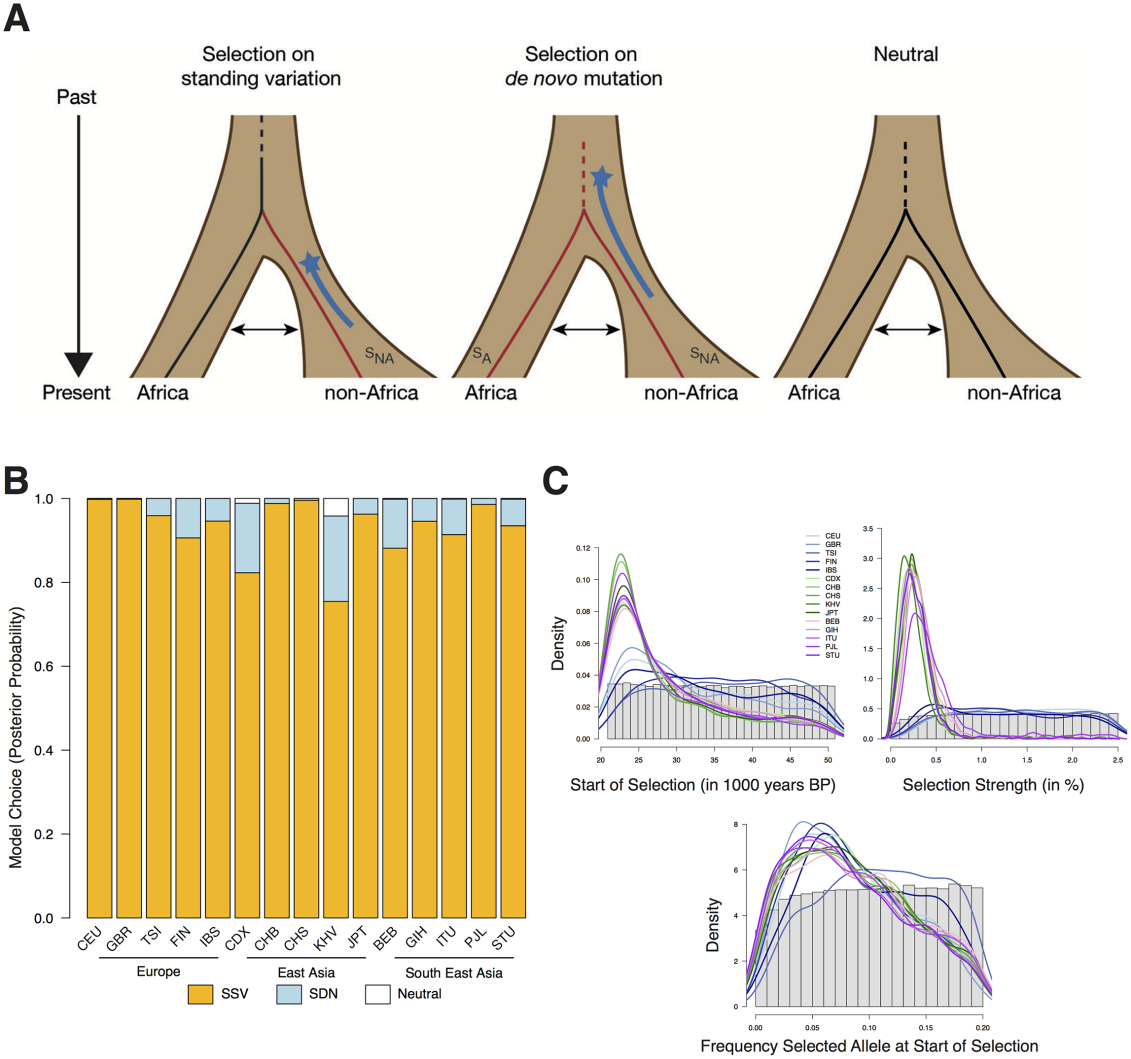
ABC analysis. **(A)** Graphical representation of the three models (SSV, SDN, NTR) and their associated parameters. Birth of the allele and start time of selection are shown by black and red lines, respectively. The range of the prior distribution for time of selection start is depicted by a star and a blue line. A double headed arrow indicates population migration. **(B)** Posterior probabilities for each model and population. **(C)** Prior distribution of each parameter as a histogram. Posterior distribution of the SSV model parameters as a line for each population.

The combination of unusually high F_ST_ values with ordinary LD patterns suggests that this locus evolved under recent, local positive selection but possibly not under a classical hard selective sweep. We formally evaluated this possibility using an Approximate Bayesian Computation (ABC) approach, which allows us to assess the probability of different evolutionary models and their associated parameters [46]. In the ABC analysis we used the summary statistics XP-EHH [44], Fay and Wu’s H [47], Tajima’s D [48], F_ST_ [49] and derived-allele-frequency, for different sections of the *TRPM8* locus (Methods) and as in [7, 50], to differentiate between three models: selection from standing variation (SSV), selection from a *de novo* variant (SDN), and a neutral model (NTR) (Fig 3A).

We have high power to identify the correct evolutionary model (the fraction of correctly assigned simulations is 96% for SDN, 81% for SSV, and 96% for NTR) with high sensitivity and specificity (S2 Table). Across all populations, the ABC results consistently favor the SSV model (Fig 3B). Bayes factors (Bayesian measure of confidence) range from 4.6 to over 500 (Table 3), representing strong to decisive evidence for the SSV model [51]. Only in KHV (2^nd^ most southern non-African population) the model choice result is inconclusive, although the SSV model still has the strongest support (Fig 3B). Interestingly, the support for the SSV model correlates moderately (almost significantly) with latitude (Pearson correlation r = 0.49, p = 0.06) because the signatures of selection are stronger at higher latitudes, as expected if the selective advantage of the T allele grew with latitude.

**Table 3:** ABC results of the SSV model for each population. Bayes factor (measure of confidence) and the resulting posterior probability (Post. Prob.) for the SSV model in each population, ordered by latitude. t_0_: time when selection starts; S_NA_: selection strength in non-African population; f_sel_: frequency of allele at selection start. The median of the posterior distribution of each inferred parameter is shown together with its 95% confidence interval (2.5%–97.5%).

Population	Bayes Factor	Post. Prob.	t_0_ (in years)			S_NA_ (in %)			f_sel_ (in %)		
			median	2.5%	97.5%	median	2.5%	97.5%	median	2.5%	97.5%
FIN	9.6	0.906	35055	22052	49881	1.238	0.304	2.430	0.078	0.010	0.189
GBR	588.3	0.998	29783	21384	49231	1.352	0.333	2.456	0.075	0.012	0.182
CEU	474.8	0.998	31390	21311	49593	1.453	0.346	2.425	0.080	0.012	0.187
TSI	23.3	0.959	36789	22088	50000	1.418	0.304	2.446	0.111	0.018	0.194
IBS	17.5	0.946	32558	21520	49666	1.209	0.250	2.409	0.090	0.012	0.191
CHB	81.8	0.988	24,529	21067	47771	0.270	0.045	0.693	0.081	0.008	0.191
JPT	25.8	0.963	25509	21103	48685	0.269	0.050	0.734	0.080	0.008	0.193
PJL	70	0.986	25017	21101	48055	0.378	0.109	2.100	0.077	0.006	0.192
BEB	7.4	0.882	26887	21118	48393	0.293	0.075	0.713	0.082	0.006	0.193
GIH	17.4	0.946	26,298	21122	48234	0.314	0.087	0.836	0.079	0.006	0.192
CHS	220.9	0.996	24407	21047	47586	0.271	0.049	0.711	0.079	0.007	0.189
CDX	4.6	0.823	26438	21088	47862	0.234	0.033	1.103	0.075	0.005	0.187
ITU	10.6	0.914	26,297	21100	48424	0.249	0.041	0.991	0.073	0.005	0.189
KHV	3.1	0.755	26399	21110	48452	0.204	0.025	1.041	0.075	0.005	0.186
STU	14.4	0.935	26024	21097	48491	0.249	0.041	0.009	0.071	0.005	0.188

Data from prehistoric Eurasians indicate that rs10166942 had reached appreciable frequency at least 3,000 years ago. It is thus possible that selection ceased sometime in the past. We evaluated this possibility with an additional ABC model selection analysis with *halted SDN* and *halted SSV* models, where the allele became neutral 3,000 years ago. Power to distinguish these two models is similar to the power to distinguish the original SDN and SSV models (S4 Table). This analysis supports the halted SSV model over the halted SDN and NTR models, with similar posterior probabilities and Bayes factors as above (S10 Fig; S5 Table). We note, however, that there is very little power to distinguish the original and halted SSV models (or the original and halted SDN models; S6 Table), as expected given their extreme similarity in signatures of selection (Table 3 and S5 Table). In any case, in all our analyses an SSV model receives stronger support than neutrality or SDN models.

The ABC framework allows estimation of the parameters of the SSV model (Table 3), although these always have large confidence intervals so median point estimates should be taken with caution. Because the ABC analyses provide no evidence for selection ceasing, we report the estimates based on the original SSV model. We infer that selection started about 26,000 years ago on an allele that was at a moderate frequency (the estimate, 7.5%, is close to its current frequency in western Africa) (Table 3) and was moderately favorable in Asia (s_Non-Africa_ = 0.28%). In Europe, we could not confidently infer the strength of selection as this parameter’s posterior distribution is quite flat (Fig 3C). This is because selection coefficients higher than 0.5 lead to almost identical summary statistic distributions (S11 Fig). However, selection strength was likely higher than 0.5 in European populations (posterior probability = 0.88), whereas in Asian populations there is little support for such high selection (posterior probability = 0.12). Together, the ABC results provide strong evidence for positive selection on neutral standing variation in all non-African populations, albeit with different selection intensities in different human groups.

## Discussion

Here we present evidence that the derived T allele of rs10166942 in *TRPM8* rose in frequency due to positive selection in a latitude-related manner. We note that while rs10166942 T is the most likely target of selection, we cannot discard that selection targeted an unknown, strongly linked allele–but this should not substantially affect our inferences. The SNP shows unusually high levels of population differentiation–it is among the 0.02% most differentiated alleles between the Yoruba and Finnish populations. Although there is a distinctive signature of high LD in the region in non-Africans, the patterns do not show clear evidence of an incomplete, hard sweep of positive selection. In fact, we infer that the derived T allele appeared in Africa and segregated neutrally, and only after the out-of-Africa migration did moderate positive selection raise the standing T allele frequency in non-African populations. ABC parameter inferences have large confidence intervals, but our point estimates indicate that selection began about 26,000 years ago, incidentally coinciding with the last glacial maximum around 26,500 years ago [52]. According to our results, selection was moderate in Asian populations and probably stronger in Europeans. This agrees with data on prehistoric humans, which indicates that rs10166942 was already at high frequency over 3,000 years ago.

Latitude, with or without temperature, predicts the rs10166942 allele frequency better than population history (the full phylogeny for PGLS, pairwise differentiation for GLMM) in both the 1KGP and SGDP datasets. Together with the F_ST_ signatures and ABC inferences, this suggests positive selection along a latitudinal cline raising the frequency of the rs10166942 T allele. We note, however, that even under comparable environmental pressure for one factor, alleles do not necessarily reach similar frequencies across populations, as many other factors differ and contribute to the overall allele-frequency. In fact, while the latitudinal cline is significant latitude and frequency do not correlate perfectly, so additional environmental factors may be at play (perhaps in Asian populations; Fig 1, S6 Fig).

TRPM8 is the only known receptor to mediate the perception of moderate cold temperature in humans (reviewed in [10]), and it has been shown to mediate the adaptive reduction of cold sensitivity in two different hibernating rodents [21]. Thus, it is likely that cold temperatures in northern latitudes were the driver of positive selection in this locus. While the precise functional effect of rs10166942 remains unknown, in large part due to the difficulties associated with studying *TRPM8* expression (see Results), the SNP falls 1kb upstream of the gene and has been predicted to have a regulatory role [22]. It is thus possible that variation in rs10166942 affects expression levels of *TRPM8*, which in turn affects cold sensation. The fact that overall current average temperature is a weaker predictor of allele frequency than latitude could be due to the considerable fluctuations of temperature over time (here, thousands of years) and the fact that the recorded data (monthly averages) is not particularly informative for long-term selective pressures. Latitude is strongly correlated with numerous other aspects of climate and is likely a good proxy for the long-term effects of climate in each of the human populations analyzed, perhaps even better than current temperature. It remains possible that other unknown functions of TRPM8 have mediated the allele frequency change, for example on the gastrointestinal system as discussed above [22].

Migraine is a debilitating neurological disorder that affects millions of people worldwide [53], and rs10166942 is among the most strongly associated SNPs with migraine risk genome-wide [23–26]. While several non-genetic traits increase the individual risk of migraine, notably being of middle age, female, suffering high stress levels, and having a low socio-economic status [54, 55], genetics play an important role. In fact, migraine is a highly heritable (34%–57% heritability [56]) yet polygenic disease [25]. Given the association between the rs10166942 C allele and low risk of migraine, the adaptive local rise in frequency of the T allele (due to direct positive selection or linkage to a selected site) could have contributed, to some extent, to differences in migraine prevalence in certain human groups. This agrees with epidemiological data: according to the World Health Organization, migraine shows low prevalence in Africa, highest prevalence in Europe, and intermediate prevalence in the Asian countries at intermediate latitudes among the two [53, 57]. In fact, migraine prevalence correlates with the evidence of positive selection and the frequency of the T allele: DAF at rs10166942 shows a positive correlation with migraine prevalence (Pearson’s rho = 0.61), although the correlation is not significant (P-value = 0.11) perhaps because we have comparable genetic [34] and migraine [57] data for only eight countries (S7 Table). Biases in disease reporting can strongly affect prevalence differences among countries, and with them this correlation result. But in the USA migraine prevalence has consistently been shown to be higher for European-Americans than African-Americans after non-genetic confounding factors are accounted for [57, 58]. Thus, while the putative influence of rs10166942 in migraine risk is moderate, and additional factors are likely at play, local adaptation in *TRPM8* may have contributed to modify, by yet unknown molecular mechanisms, pain-related phenotypes in human populations.

## Materials & methods

### The rs10166942 T allele

The variant rs10166942 is located ~1 kb upstream of the *TRPM8* gene. We used a combination of bioinformatics tools to investigate possible functional effects of rs10166942 and it neighboring variants in high linkage disequilibrium (LD). We explored the predicted effects on protein sequence using variant effect predictor (VEP) [59], focusing on the non-synonymous and splice-site SNPs, as well as indels annotated in the 1KGP. We explored effects on gene expression using Regulome DB annotations [60], GTEx data [61] and basal root ganglion RNA-Seq data (kindly provided by G. Gisselmann) [62].

### Modern genomes

To investigate the patterns of genetic diversity of *TRPM8* we used genome-wide genotype data from the 1KGP phase III [34]. African ancestry: ESN (Esan in Nigeria), GWD (Gambian (Mandinka) in Western Divisions in Gambia), YRI (Yoruba in Ibadan, Nigeria), LWK (Luhya in Webuye, Kenya), MSL (Mende in Sierra Leone), ASW (African Ancestry in Southwest USA), ACB (African Caribbean in Barbados); European ancestry: GBR (British from England and Scotland), CEU (Utah Residents, USA, with Northern and Western European ancestry), FIN (Finnish from Finland), TSI (Toscani in Italia), IBS (Iberian Populations in Spain); East Asian ancestry: CHS (Southern Han Chinese), CHB (Han Chinese in Beijing, China), JPT (Japanese in Toyko, Japan), CDX (Chinese Dai in Xishuangbanna, China), KHV (Kinh in Ho Chi Minh City, Vietnam); South Asian ancestry: BEB (Bengali in Bangladesh), GIH (Gujarati Indians in Houston, USA), ITU (Indian Telugu in the UK), PJL (Punjabi in Lahore, Pakistan), STU (Sri Lankan Tamil in the UK). The American populations from the 1KGP have recent admixture with Europeans [63], and thus are not suited for our analysis and were excluded. Across the 22 populations the lowest sample size is 61 (ASW), so to minimise power differences among populations we randomly down-sampled each population to 61 unrelated individuals.

We also used the genetic data from the 142 populations of the SGDP project dataset, together with their meta-information (including geographic location) [41]. For the geographic location, in the southern hemisphere we used the absolute value of the latitude. Most populations have high coverage whole-genome sequencing data for two representative individuals, so we used two individuals from each ‘Panel C’ population with a sample size of at least two (110 populations).

### Early Eurasian genomes

Ancient genomes were used to infer the frequency of rs10166942 T in different pre-historic human populations. The genotype data from ancient paleo-eskimo individuals from the Saqqaq culture [64] were obtained from the Danish bioinformatics center. Data on early Europeans [42] was downloaded from the Reich lab webpage. We transformed the binary eigenstrat file to a vcf using eigenstrat2vcf.py and extracted the genotype information for rs10166942. Age information was extracted from Supplementary Data 1 in [42]. After filtering, we were able to genotype 79 ancient individuals for rs10166942. These individuals lived in Eurasia 3,000 to 8,500 years ago and represent three different ancestry groups: Hunter-Gatherers (8 individuals), Early Farmers (33 individuals), and Steppe pastoralists (38 individuals).

### Origin of the rs10166942 T allele

We inferred the likely place of origin for the rs10166942 T allele by analysing haplotypes carrying the derived T allele, as levels of linked variation should be highest in the population closest to the one where it appeared. Since no homozygous T/T individuals are present in several of the 1KGP populations, we relied on the phased haplotypes across the 65kb region of interest. We calculated pi after removing derived haplotypes with evidence of recombination with ancestral rs10166942 C allele (S1 Table).

### Latitude and temperature estimates

In order to investigate the correlation of allele frequencies with latitude and temperature, we jointly analysed genetic, latitude, and temperature information. For modern humans, we estimated the absolute latitude of the location of each population according to Wikipedia and Google Maps (Table 1). The CEU population, of central European ancestry, was assigned the coordinates of Brussels. For early modern humans, latitude information was extracted from Supplementary Data 1 in [42] and updated when necessary (e.g., some individuals lacked geographic coordinates or had problems with the longitude/latitude information).

Temperature time series information was extracted for 2001–2010 from a 0.5°x0.5° grid matrix assembled at the Climate Research Unit of the University of East Anglia (version 3.23; [65]). Data is available since 1960, but we used only the time series from 2001–2010 to guarantee comparable and high-quality estimates across populations. Using the geographic coordinates of each population we extracted annual mean temperatures.

### Phylogenetic Generalized Least Squares (PGLS)

To investigate to what extent shared ancestry, latitude and temperature predict rs10166942 T allele frequency in each population we used two different linear models. We first used a PGLS analysis [40], which can account for the full phylogenetic signal (the population relationships) present in our data [39]. The response variable is the mean derived allele frequency of the rs10166942 T allele per population. We first conducted a null/full model comparison. The null model contains only the shared ancestry information (the ‘phylogeny’); here, we used the full pairwise F_ST_ matrix averaged across all positions polymorphic in that particular population pair. Following Weir and Cockerham, we calculated the genome-wide average F_ST_ between two populations as the “ratio of averages” (equation 10 in [49]). A neighbor-joining (NJ) tree was calculated using a matrix of the pairwise F_ST_ values with the R package *ape* [66], and rooted using ‘mid-point’ rooting with *Archaeopteryx* [67]. The full model includes additional predictor variables: *latitude* and annual mean *temperature*. In order to achieve convergence of the model we z-transformed each predictor. We excluded populations one at a time and compared the model estimates derived from the subsets with those obtained from the full data set, which revealed the model to have good stability. We assessed for the full model whether the assumptions of normally distributed and homogenous residuals were fulfilled by visual inspection of a QQ-plot of the residuals and residuals plotted against fitted values [68], which revealed no issues with these assumptions. As an overall test of the effect of the two test predictors (*latitude* and annual mean *temperature*), we compared the fit of the full model with that of the null model [69] using a likelihood ratio test [70].

We then performed a multi-model inference [71] to compare the null model and all possible models that could be constructed with the two test predictors (four models in total). To quantify the relative performance of each model, we used Akaike’s Information Criterion (AIC, corrected for small samples) as a measure of model fit penalized for model complexity, and determined Akaike weights as a measure of the support a model received compared to all other models in the set [71]. In practice, we use the Akaike weights to derive the 95% best model confidence (comprising the truly best model in the model set with a probability of 0.95) and also to determine Akaike weights for the individual predictors by summing the Akaike weights of the models comprising them. To infer the overall relevance of predictors in the model set we determined whether the null model was included in the 95% best model confidence set [72]. The analysis was conducted in R [73] using the function pgls of the package caper [74].

### Generalized Linear Mixed Models (GLMM)

To be able to analyze both the 1KGP and the SGDP datasets (which has small sample size for a large number of populations, so allele frequencies cannot be estimated) we also used a GLMM [75] fitted with binomial error structure and logit link function [76]. This model conceptually corresponds to a regression; however, it allows more flexibility with regard to the distribution of the response (e.g., normality and homogeneity of the residuals are not necessarily required), and it also allows us to effectively control for non-independence of the data due to multiple observations of the same populations or individuals [75]. The response variable is the genotype of rs10166942 in each individual, in a 2-column-reponse-matrix (the derived and the ancestral allele counts). For the modern human genetic data, shared ancestry was controlled by adding as an additional fixed effect the genetic distance between each population and YRI, measured as the genome-wide average F_ST_. Population identity was included as a random effect in the model, to account for random genetic drift. We further included a random effect per individual to account for the non-independence of the ancestral and derived allele counts. The model that includes all these effects is the null model.

To test for the effects of *latitude* and the annual mean *temperature* we included them as test predictor variables with fixed effects. In the analysis of the early Europeans, we added *age* as a further test predictor variable. For the comparison among models (multi model inference [71]) we considered the null model and all possible models that could be constructed with the two test predictors, totaling four models (eight in the early European analysis). We assessed model stability as in case of the PGLS, which revealed the model to have good stability (S3 Table). Overdispersion was no issue (dispersion parameter of the full model in the 1KGP: 0.97 and the SGDP: 0.67). The models were fitted in R [73] using the library ‘lme4’ [77].

### Signatures of local adaptation

Local adaptation on a single variant can lead to a rapid rise in the frequency of the positively selected allele, resulting in strong population differentiation (measured for example by F_ST_) between the population(s) with positive selection and those without it. We calculated per SNP F_ST_ with a custom *perl* implementation of the Weir and Cockerham estimator [49] for each pairwise population comparison.

The allele under positive selection will rise in frequency together with its background haplotype, raising the frequency of linked alleles. When the favoured allele is young (e.g., under a classic selection from a de-novo mutation model (SDN) hard sweep model), this results in a signature of extended haplotype homozygosity. To test for such signature, we calculated iHS [45] and XP-EHH [44] using *selscan* with default parameters [78]. For iHS, we used SNPs with derived allele frequencies higher than 5% and lower than 95%. For XP-EHH, we used SNPs with derived allele frequency higher than 5% in the test population. These filters follow previously established methods [79] and prevent signatures of extended LD to be broken by rare variants, while still obtaining XP-EHH values for derived alleles fixed or nearly fixed in the *test* population. For both analyses, only sites with a high confidence inferred ancestral allele were used (part of 1KGP genotype files). Recombination was estimated using the genetic map from HapMap Project, Phase 2 [80].

All three statistics were calculated genome-wide, and P-values for SNPs of interest were calculated based on the empirical distribution. Since both tests are sensitive for positive selection, the tail of the empirical distribution is enriched for the targets of positive selection. Our analysis is hypothesis-driven for the migraine risk allele in rs10166942, and, thus, no correction for multiple testing is required.

### Approximate Bayesian Computation analysis

To infer the selective history of the gene, we used an Approximate Bayesian Computation (ABC) approach, which allows us to assess the probability of different evolutionary models and their associated parameters [46]. Following [7, 50], we compared the genomic observations to simulations under three models with parameters drawn from uniform (U) prior distributions. These models are: (I) SDN, where the selected allele appeared as a single copy between 60,000 and 30,000 years ago (t_mut_~U(30,000, 60,000 years ago)) and was immediately advantageous with a selective coefficient that was allowed to differ between the African (s_A_~U(0,1.5%)) and the non-African (s_NA_~U(0.5,5%)) populations; (II) selection on standing variation (SSV), where a previously neutral allele at a given starting frequency (f_sel_~U(0,20%)) became positively selected (s_NA_~U(>0,5%)) in the non-African population after the out of Africa migration and before the European-Asian split (51,000 to 21,000 years ago; t_mut_~U(21,000, 51,000 years ago)); (III) fully neutral model (NTR), where the allele appeared as in the SDN model (t_mut_~U(30,000, 60,000 years ago)) but was completely neutral.

We ran one million simulations for each selection model and 100,000 simulations for the neutral model using msms [81]. Each simulation comprised a stretch of 185 kb with 122 chromosomes of an African (population 1) and a non-African (population 2) population. Human demographic parameters followed the model inferred by Gravel et al. [82], and in each simulation we analyzed the African population with one non-African population (in Europe or Asia). To simulate the recombination hotspots across the locus, we simulated extended regions with a length that corresponded to the local increase in recombination rate above the baseline recombination rate (S12 Fig). These regions were then removed before calculating summary statistics, such that they contribute recombination events but not mutation events to the data. The baseline recombination rate was the mean recombination rate across the locus excluding the peaks, based on a merged map from several 1KGP populations (S12 Fig).

For the ABC inference we used five summary statistics: XP-EHH [44], Fay and Wu’s H [47], Tajima’s D [48], F_ST_ [49] and derived-allele-frequency. XP-EHH and F_ST_ were calculated between YRI and the studied population. We calculated the LD based statistic XP-EHH on the selected allele using the entire simulated region. We calculated the statistics Fay and Wu’s H, Tajima’s D, and average F_ST_ (across SNPs in a section) in both simulated populations on two separate sections: the first section was the central ~65 kb part (since the genomic data shows strong population differentiation across 65 kb), and the second section were the combined flanking regions, together 120 kb long. We also used the allele frequency of the selected site in the African and non-African population and its F_ST_.

As in the genomic data, for the XP-EHH statistic we required the variant investigated to have a derived allele frequency > 5% in the *test* non-African population. The absence of a long haplotype associated with the derived allele (XP-EHH) in the presence of strong population differentiation is an important attribute to differentiate between the SDN and the SSV model [83–85]. Thus, we used only simulations where XP-EHH could be calculated, which biased minimally the previously uniform prior.

All summary statistics were calculated in the same way for the simulations and the real data–where rs10166942 was used as a proxy for the selected site. The demographic history follows the [82] model. African demography was based on YRI, all European populations (CEU, GBR, TSI, FIN, IBS) were simulated under the inferred European (CEU) demography, and all Asian populations (CDX, CHB, CHS, KHV, JPT, BEB, GIH, ITU, PJL, STU) under the inferred East Asian (CHB/JPT) demography. The ABC analysis was performed using the ABCtoolbox on BoxCox and PLS transformed summary statistics (following recommendations for ABCtoolbox) [86] retaining the top 1,000 simulations matching our observation. We used the first five PLS components as they carried most information for each parameter (S13 Fig). The PLS transformed statistics differentiate between the different models and capture the variation observed (S11 Fig), rendering them well-suited for the inference.

We performed an additional ABC inference considering a halted SDN and a halted SSV model (with all parameters as above, with the only exception that selection ceased 3,000 years ago). Both power estimates and model selection were performed as described above. Lastly, we also performed an ABC analysis with the four selection models (SDN, partial SDN, SSV and partial SSV) to test our power to discriminate among them.

## Supporting information

S1 Fig Tissue expression of *TRPM8* according to GTEx dataset. Known eQTLs are absent in the region (RegulomeDB [60]), although the restricted expression of the gene may hamper their identification. Because the gene is also expressed in prostate according to GTEx [61], we investigated if rs10166942 affects expression in this tissue type. rs10166942 was not included on the Illumina 2.5 M SNP array used to genotype the majority of individuals in this cohort, so we used instead available tagging SNPs in high LD (in FIN; rs6431648 r^2 =^ 0.73, rs4663990 r^2 =^ 0.6, and rs917435 r^2 =^ 0.6). Using genotypes and prostate RNA-Seq data from 62 individuals from the GTEx cohort we were unable to detect allele-specific differential expression of the whole gene and any of the exons, for any of the three tagging SNPs considered. We note that we were unable to analyze *TRPM8* expression in available basal root ganglion RNA-Seq data (kindly provided by G. Gisselmann) from 21 pooled human samples (all European ancestry) [62] because out of 20.1 million 75-bp reads, only 187 map to the 5,621 bp transcript RefSeq NM_024080.4 (at ~2x average read depth).
(PDF)

S2 Fig Protein-coding variants located in *TRPM8*. Three variants in close proximity to rs10166942 (all with intermediate to low LD) are non-synonymous (rs7593557 S419N r^2 =^ 0.28, rs13004520 R247T r^2 =^ 0.06, rs17868387 Y251C r^2 =^ 0.06), but they all fall in the N-terminal domain of TRPM8 and are unlikely to affect protein function. There are no indels that affect the open-reading frame of *TRPM8*.
(PDF)

S3 Fig Pairwise differences among haplotypes carrying the derived rs10166942 T allele. Distribution of pairwise differences of each haplotype carrying the rs10166942 derived T allele (*derived haplotype*) with all other derived haplotypes within a population. We show one representative population for each continent: YRI (Africa), CHB (East Asia), GIH (South Asia), and FIN (Europe). The marked boxplots (orange; median > 10) indicate haplotypes putatively affected by recombination with the ancestral haplotype (carrying the rs10166942 ancestral C allele). These haplotypes have not only unusually large distances to other derived haplotypes, but the alleles contributing to these differences are by large present in the ancestral background (S4 Fig).
(PDF)

S4 Fig Proportion of variants present on derived haplotypes likely due to recombination. Y-axis shows, of all the variable sites (with median pairwise difference of 10 and higher, marked in S3 Fig) present on the *derived* haplotypes (carrying the rs10166942 derived T allele), which proportion of the alleles are also present in the *ancestral* haplotypes (carrying the ancestral rs10166942 C allele). The observed high proportion indicates that these derived haplotypes most likely arose as a result of recombination with the ancestral haplotype. All populations with at least one allele with a median pairwise count above 10 are shown (number of alleles (N) in parenthesis).
(PDF)

S5 Fig Neighbor-Joining tree for the 1KGP populations, based on the genome-wide F_ST_ matrix. (PDF)

S6 Fig SGDP population overview. Map showing the geographic origin of each population and its rs10166942 T allele count for the two individuals sampled (additional information Supplemental Dataset 1).
(PDF)

S7 Fig Latitude and age of each pre-historic European considered. Colour indicates ancestry group: EF for Early Farmers (orange), HG for Hunter-Gatherers (blue), and SP for individuals of Steppe pastoralist ancestry (red). The genotype of the ancient individual is indicated by its symbol (. for missing data; 0 for homozygote ancestral; 1 for heterozygote; 2 for homozygote derived). The legend shows the Pearson’s correlation of the allele count with latitude within each ancestry group.
(PDF)

S8 Fig Selection signatures across the *TRPM8* locus. Empirical P-values for F_ST_ (blue circles) and XP-EHH (grey diamonds) in the extended *TRPM8* region in all populations analysed. The position of *TRPM8* is indicated by an orange bar on top, while the strongly differentiated upstream region is between the two vertical blue lines. The red circle marks the F_ST_ value and the red diamond the XP-EHH value of candidate variant rs10166942. Long dashed lines show mean P-value for F_ST_ and XPEHH (blue and grey, respectively; largely overlapping), across all protein-coding genes on chromosome 2 (ensembl GRCh37.p13).
(PDF)

S9 Fig Linkage disequilibrium across extended *TRPM8* locus. Haploview (https://www.broadinstitute.org/haploview/haploview) plots for (**A**) CHB and (**B**) FIN across a +-20 kb extended region surrounding*TRPM8*.
(PDF)

S10 Fig ABC analysis with selection halted 3,000 years ago. Posterior probabilities for each model and population.
(PDF)

S11 Fig Cloud plots of PLS transformed statistics. Scatter plots of all five PLS components used in the ABC inference for Europe (**A** & **C**) and Asia (**B** & **D**). (**A** & **B**) PLS transformed statistics for the SSV model and their correlation with the three parameters associated with the SSV model: s_time (time when selection started), s_strength NA (selection strength in non-Africa) and frequency (frequency of the allele at s_time). (**C** & **D**) The PLS transformed statistics for all three models (SDN in blue, SSV in orange and NTR in grey) and the PLS transformed observations in all non-African populations (color scheme as in Fig 1).
(JPG)

S12 Fig Recombination landscape across the *TRPM8* locus. Recombination map based on average recombination rate in two randomly chosen populations per continental group, to avoid biases due to different numbers of populations per continent (YRI, LWK for Africa; GBR, TSI for Europe; CHB, GIH for Asia). The *TRPM8* gene is between the two blue vertical dashed lines. The strongly differentiated region is between the two red vertical dashed lines. All basepairs with recombination rates higher than 5 cM/Mb (horizontal dashed line) were considered as being within a hotspot of recombination in the simulations.
(PDF)

S13 Fig RMSE plots. Information contained within each PLS component for a given parameter for all three models combined for (**A**) the European model and (**B**) the Asian model. *t*_0_ (time when selection started), s_A_ (selection strength in Africa), s_NA_ (selection strength in non-Africa), *f*_sel Africa_ (frequency of the allele at selection start in Africa), *f*_sel Non-Africa_ (frequency of the allele at selection start in non-Africa).
(PDF)

S1 Table Linked diversity. (**A**) Diversity estimates measured by means of the number of pairwise differences for all haplotypes carrying the derived rs10166942 T allele. (**B**) Same as in **A** after removing haplotypes with evidence of recombination (see Materials and methods and S3 and S4 Figs).
(DOCX)

S2 Table Power results of ABC analysis with continuous selection. Power of ABC analysis to correctly assign the model in simulations of European and Asian demography using 10,000 random samplings. TP (True Positive), FP (False Positive), and FN (False Negative).
(DOCX)

S3 Table Model stability. Full model stability estimates for each fixed and random effect in each analysis (original estimate obtained from the full data set and the range of estimates derived from omitting individuals and populations (GLMM) or populations (PGLS), one at a time). The small ranges around the original value indicate the overall good stability of the model. Based on z-transformed predictor variables for the PGLS analysis and the GLMM analysis of the SGDP data.
(DOCX)

S4 Table Power results of ABC analysis with selection ceased 3,000 years ago. Power of ABC analysis to correctly assign the model in simulations of European and Asian demography using 10,000 random samplings. TP (True Positive), FP (False Positive), and FN (False Negative).
(DOCX)

S5 Table ABC results of the halted SSV model for each population. Bayes factor (measure of confidence) and the resulting posterior probability (Post. Prob.) for the SSV model in each population, ordered by latitude. t_0_: time when selection starts; S_NA_: selection strength in non-African population (ceased 3,000 years ago); f_sel_: frequency of allele at selection start. The median of the posterior distribution of each inferred parameter is shown together with its 95% confidence interval (2.5%–97.5%).
(DOCX)

S6 Table Power results of ABC analysis to differentiate continuous selection and selection ceased 3,000 years ago. Power of ABC analysis to correctly assign the selection model in simulations of European and Asian demography using 10,000 random samplings. TP (True Positive), FP (False Positive), and FN (False Negative).
(DOCX)

S7 Table Migraine prevalence and derived allele frequency. Migraine prevalence per country gathered from Stovner et al. [57]. When multiple samplings per population were available, mean migraine prevalence or mean DAF reported. Pearson correlation between DAF and migraine prevalence: rho = 0.61 (p-value = 0.11).
(DOCX)

S1 Dataset Overview SGDP data. For each individual used from the SGDP ‘C Panel’ the ID, population, continent, rs10166942 ancestral and derived allele count, latitude, longitude and mean yearly temperature are given.
(TXT)
