# Supplementary figures and images for: Human local adaptation of the TRPM8 cold receptor along a latitudinal cline
Source: PLoS Genet. 2018 May 3;14(5):e1007298. doi: 10.1371/journal.pgen.1007298 (PMC5933706; doi:10.1371/journal.pgen.1007298)

TRPM8 Gene Expression

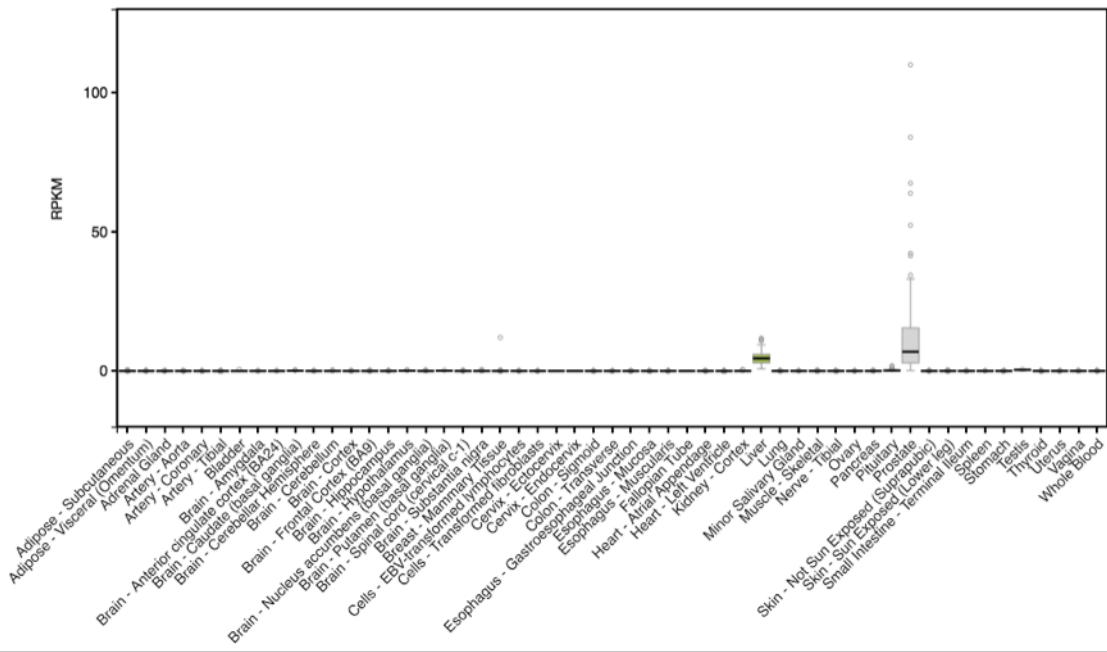

Supplement: S1 Fig — Known eQTLs are absent in the region (RegulomeDB [60]), although the restricted expression of the gene may hamper their identification. Because the gene is also expressed in prostate according to GTEx [61], we investigated if rs10166942 affects expression in this tissue type. rs10166942 was not included on the Illumina 2.5 M SNP array used to genotype the majority of individuals in this cohort, so we used instead available tagging SNPs in high LD (in FIN; rs6431648 r2 = 0.73, rs4663990 r2 = 0.6, and rs917435 r2 = 0.6). Using genotypes and prostate RNA-Seq data from 62 individuals from the GTEx cohort we were unable to detect allele-specific differential expression of the whole gene and any of the exons, for any of the three tagging SNPs considered. We note that we were unable to analyze TRPM8 expression in available basal root ganglion RNA-Seq data (kindly provided by G. Gisselmann) from 21 pooled human samples (all European ancestry) [62] because out of 20.1 million 75-bp reads, only 187 map to the 5,621 bp transcript RefSeq NM_024080.4 (at ~2x average read depth). (PDF) [file pgen.1007298.s001.pdf]

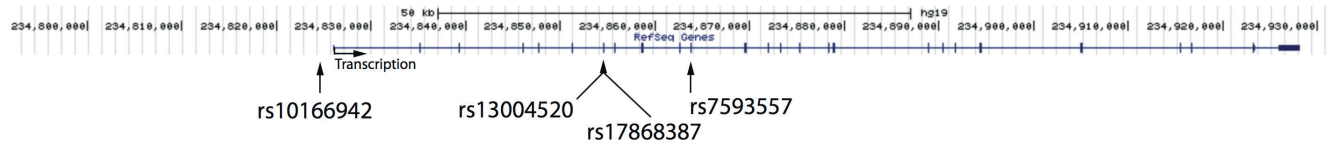

Supplement: S2 Fig — Three variants in close proximity to rs10166942 (all with intermediate to low LD) are non-synonymous (rs7593557 S419N r2 = 0.28, rs13004520 R247T r2 = 0.06, rs17868387 Y251C r2 = 0.06), but they all fall in the N-terminal domain of TRPM8 and are unlikely to affect protein function. There are no indels that affect the open-reading frame of TRPM8. (PDF) [file pgen.1007298.s002.pdf]

CHB

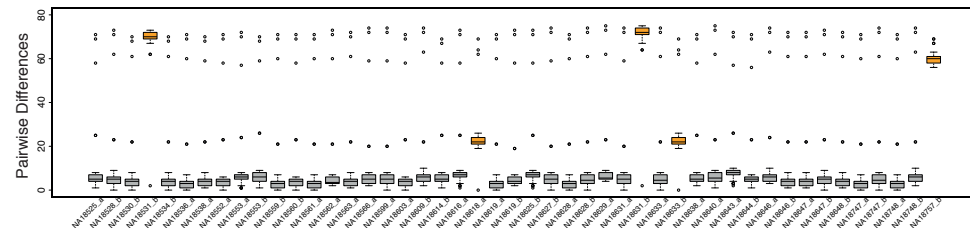

GIH

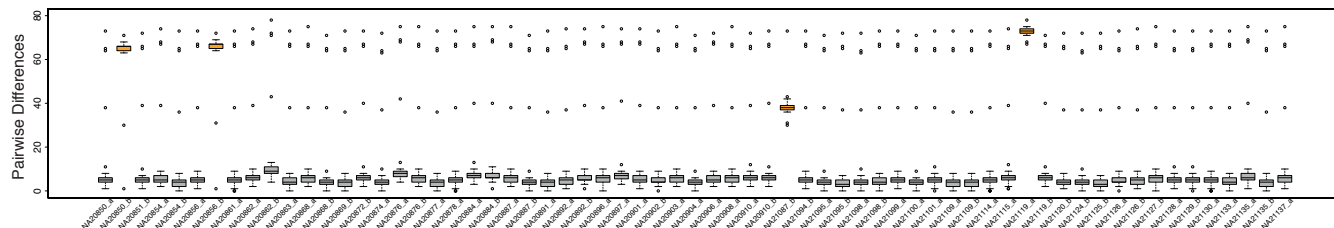

FIN

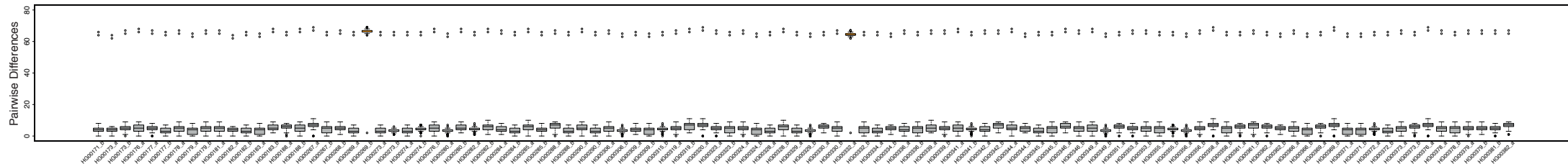

Supplement: S3 Fig — Distribution of pairwise differences of each haplotype carrying the rs10166942 derived T allele (derived haplotype) with all other derived haplotypes within a population. We show one representative population for each continent: YRI (Africa), CHB (East Asia), GIH (South Asia), and FIN (Europe). The marked boxplots (orange; median > 10) indicate haplotypes putatively affected by recombination with the ancestral haplotype (carrying the rs10166942 ancestral C allele). These haplotypes have not only unusually large distances to other derived haplotypes, but the alleles contributing to these differences are by large present in the ancestral background (S4 Fig). (PDF) [file pgen.1007298.s003.pdf]

Proportion excess pairwise diversity alleles on ancestral haplotype

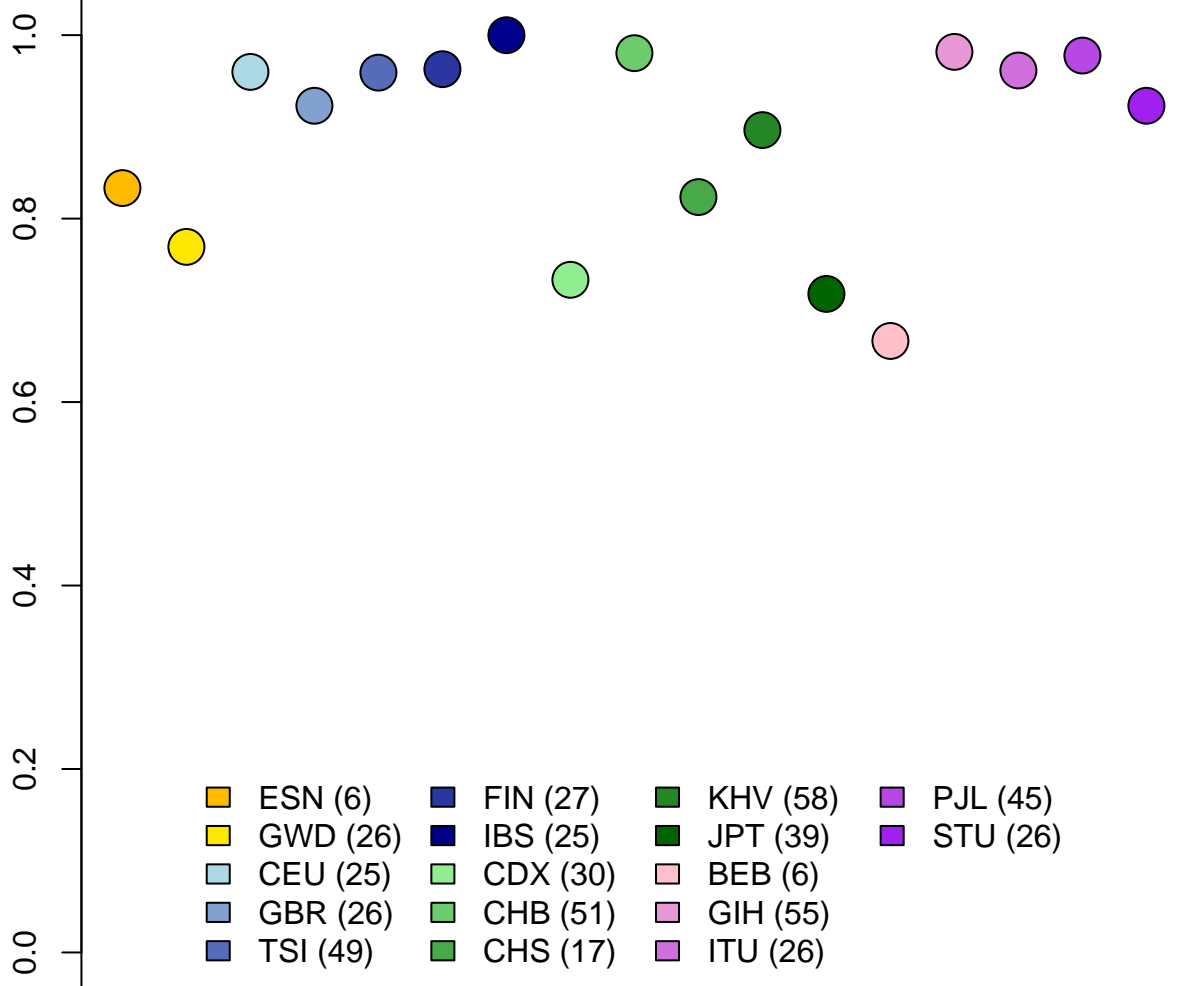

Supplement: S4 Fig — Y-axis shows, of all the variable sites (with median pairwise difference of 10 and higher, marked in S3 Fig) present on the derived haplotypes (carrying the rs10166942 derived T allele), which proportion of the alleles are also present in the ancestral haplotypes (carrying the ancestral rs10166942 C allele). The observed high proportion indicates that these derived haplotypes most likely arose as a result of recombination with the ancestral haplotype. All populations with at least one allele with a median pairwise count above 10 are shown (number of alleles (N) in parenthesis). (PDF) [file pgen.1007298.s004.pdf]

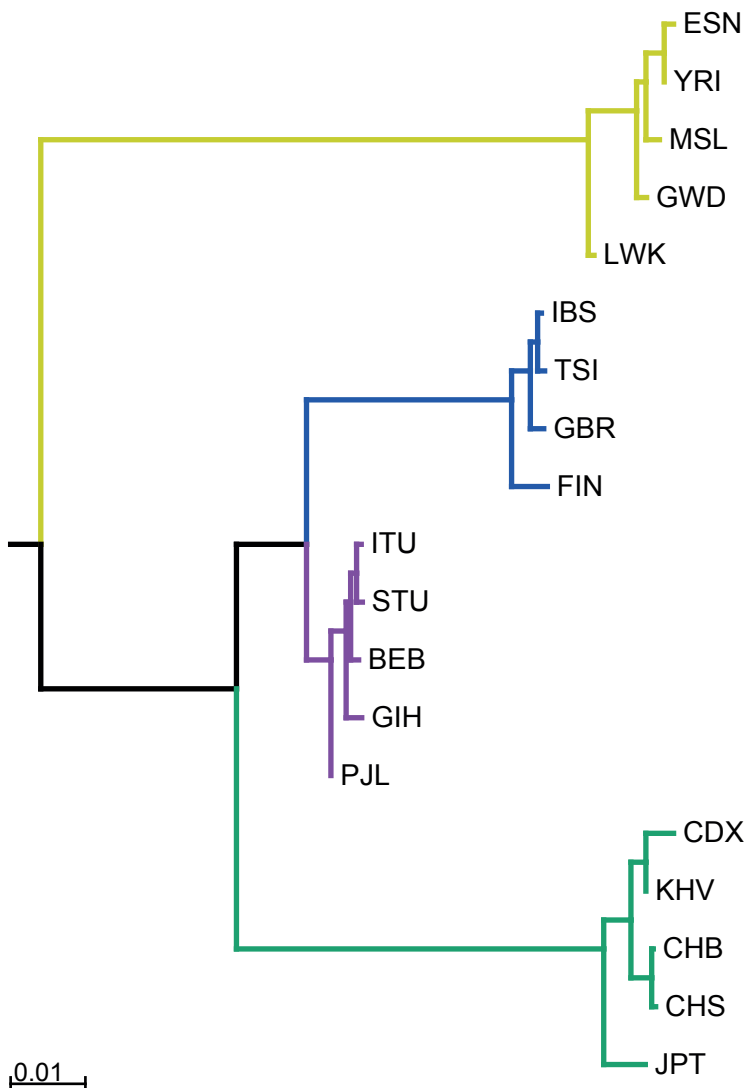

Supplement: S5 Fig — (PDF) [file pgen.1007298.s005.pdf]

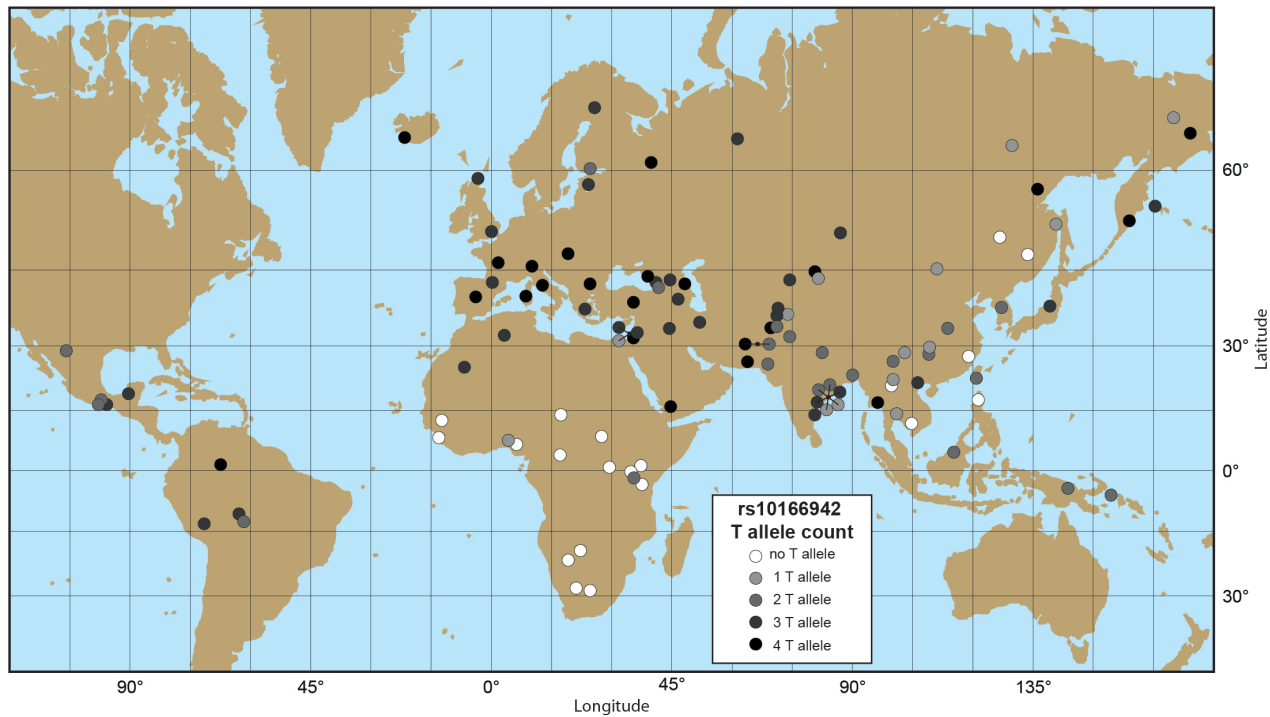

Supplement: S6 Fig — Map showing the geographic origin of each population and its rs10166942 T allele count for the two individuals sampled (additional information Supplemental Dataset 1). (PDF) [file pgen.1007298.s006.pdf]

# Genotype Migraine SNP across ancEUR

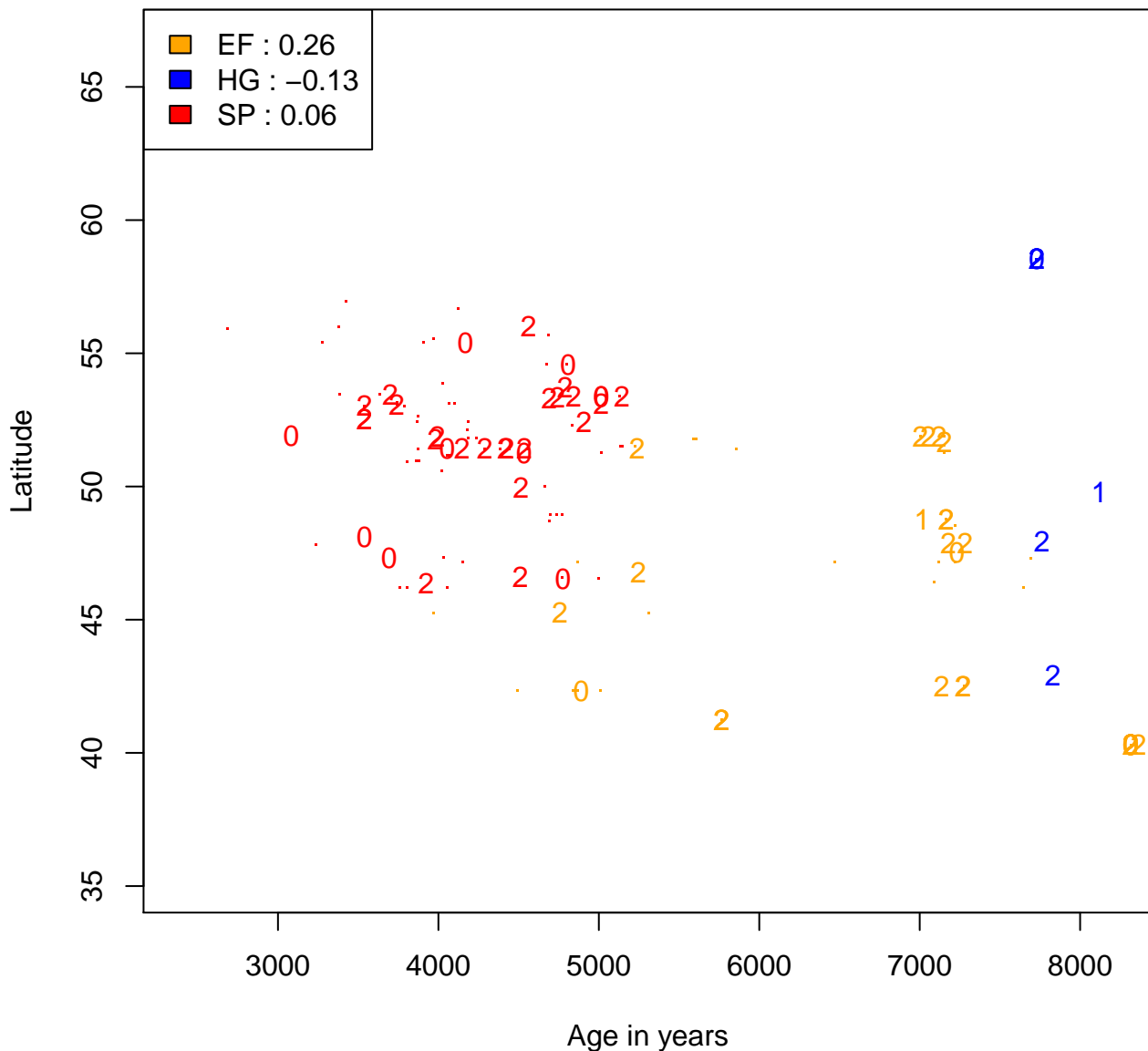

Supplement: S7 Fig — Colour indicates ancestry group: EF for Early Farmers (orange), HG for Hunter-Gatherers (blue), and SP for individuals of Steppe pastoralist ancestry (red). The genotype of the ancient individual is indicated by its symbol (. for missing data; 0 for homozygote ancestral; 1 for heterozygote; 2 for homozygote derived). The legend shows the Pearson’s correlation of the allele count with latitude within each ancestry group. (PDF) [file pgen.1007298.s007.pdf]

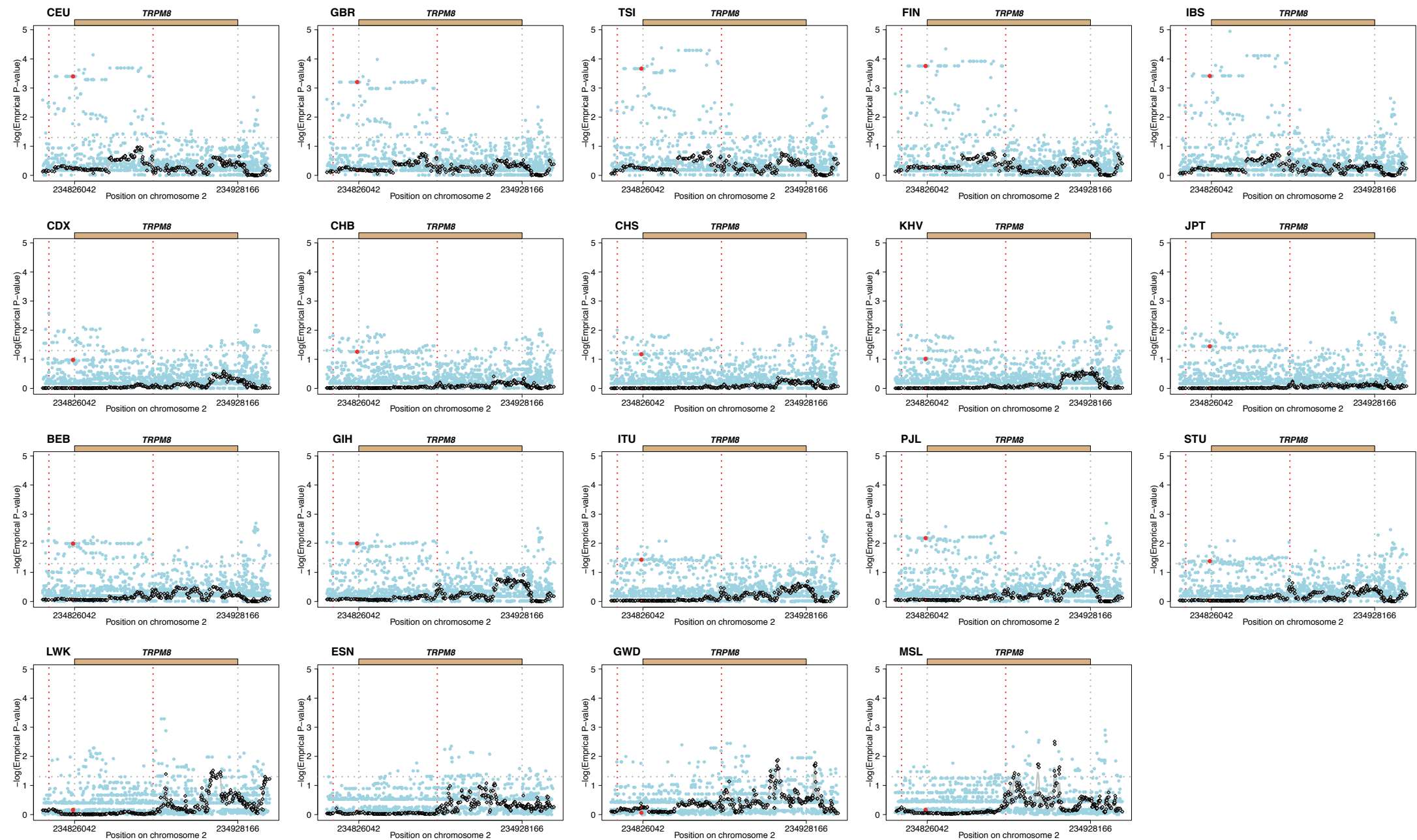

Supplement: S8 Fig — Empirical P-values for FST (blue circles) and XP-EHH (grey diamonds) in the extended TRPM8 region in all populations analysed. The position of TRPM8 is indicated by an orange bar on top, while the strongly differentiated upstream region is between the two vertical blue lines. The red circle marks the FST value and the red diamond the XP-EHH value of candidate variant rs10166942. Long dashed lines show mean P-value for FST and XPEHH (blue and grey, respectively; largely overlapping), across all protein-coding genes on chromosome 2 (ensembl GRCh37.p13). (PDF) [file pgen.1007298.s008.pdf]

A

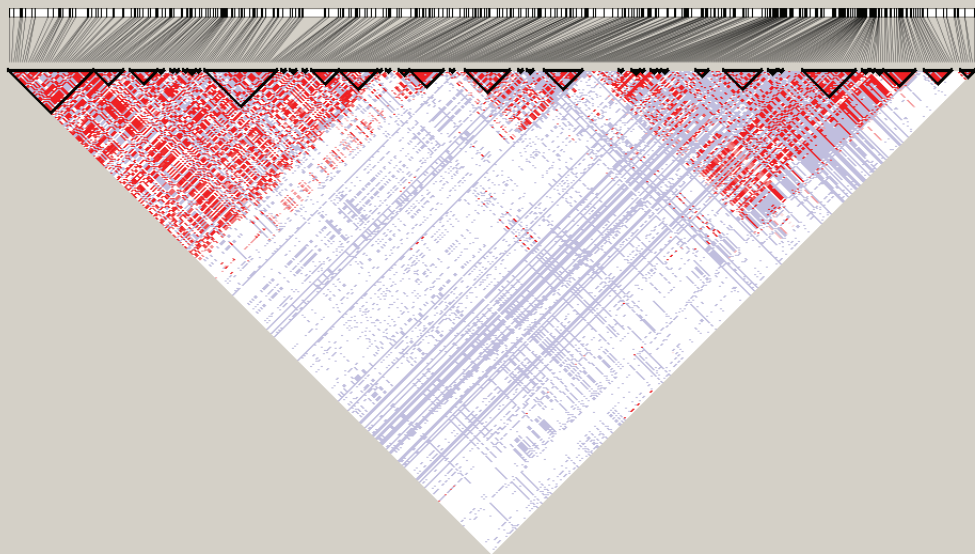

B

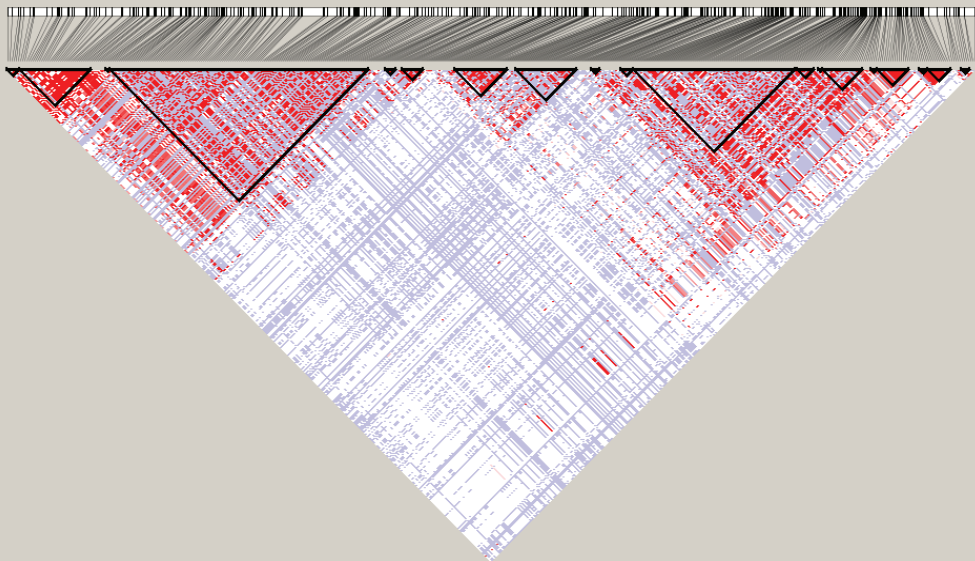

Supplement: S9 Fig — Haploview (https://www.broadinstitute.org/haploview/haploview) plots for (A) CHB and (B) FIN across a +-20 kb extended region surroundingTRPM8. (PDF) [file pgen.1007298.s009.pdf]

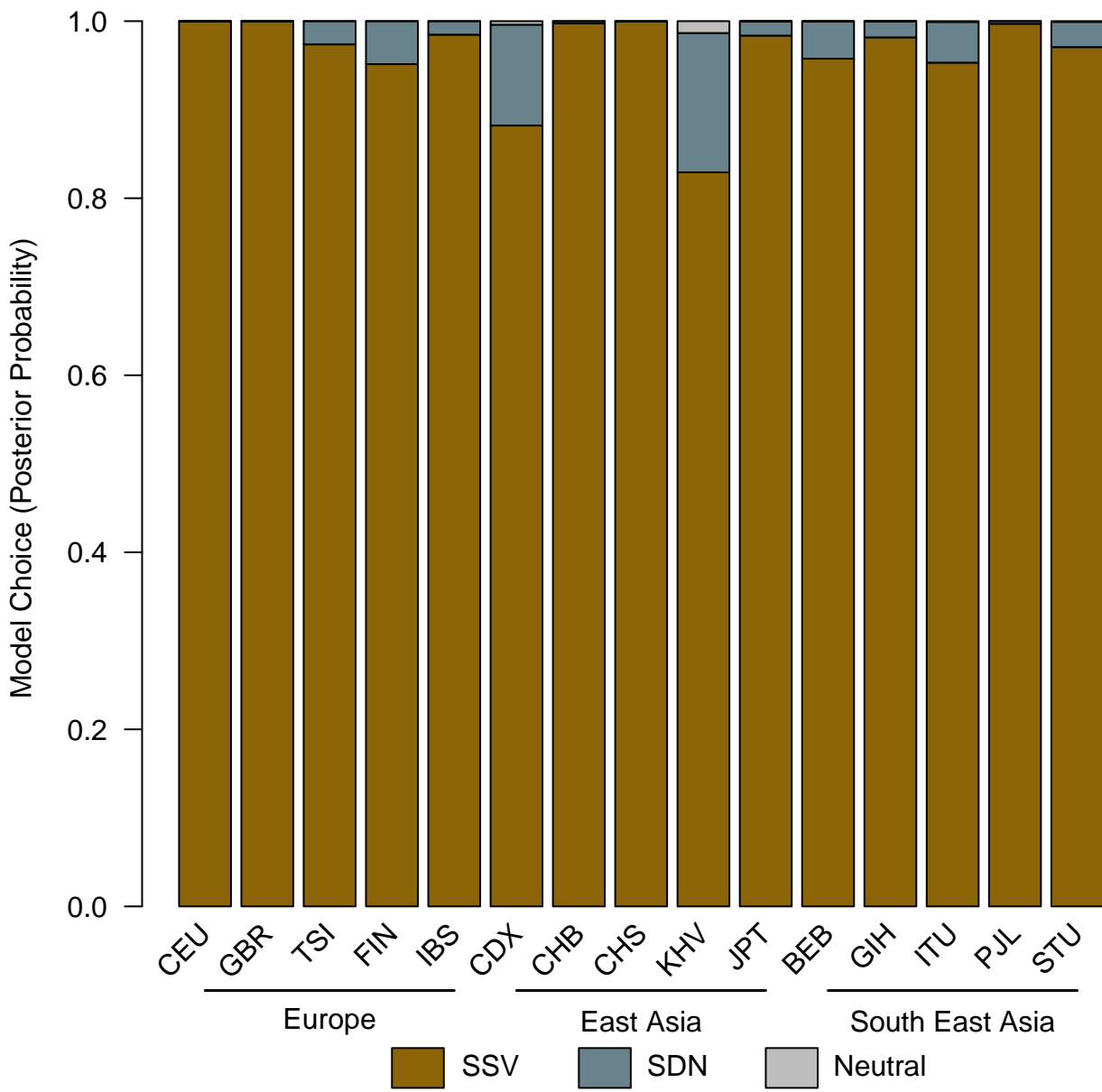

Supplement: S10 Fig — Posterior probabilities for each model and population. (PDF) [file pgen.1007298.s010.pdf]

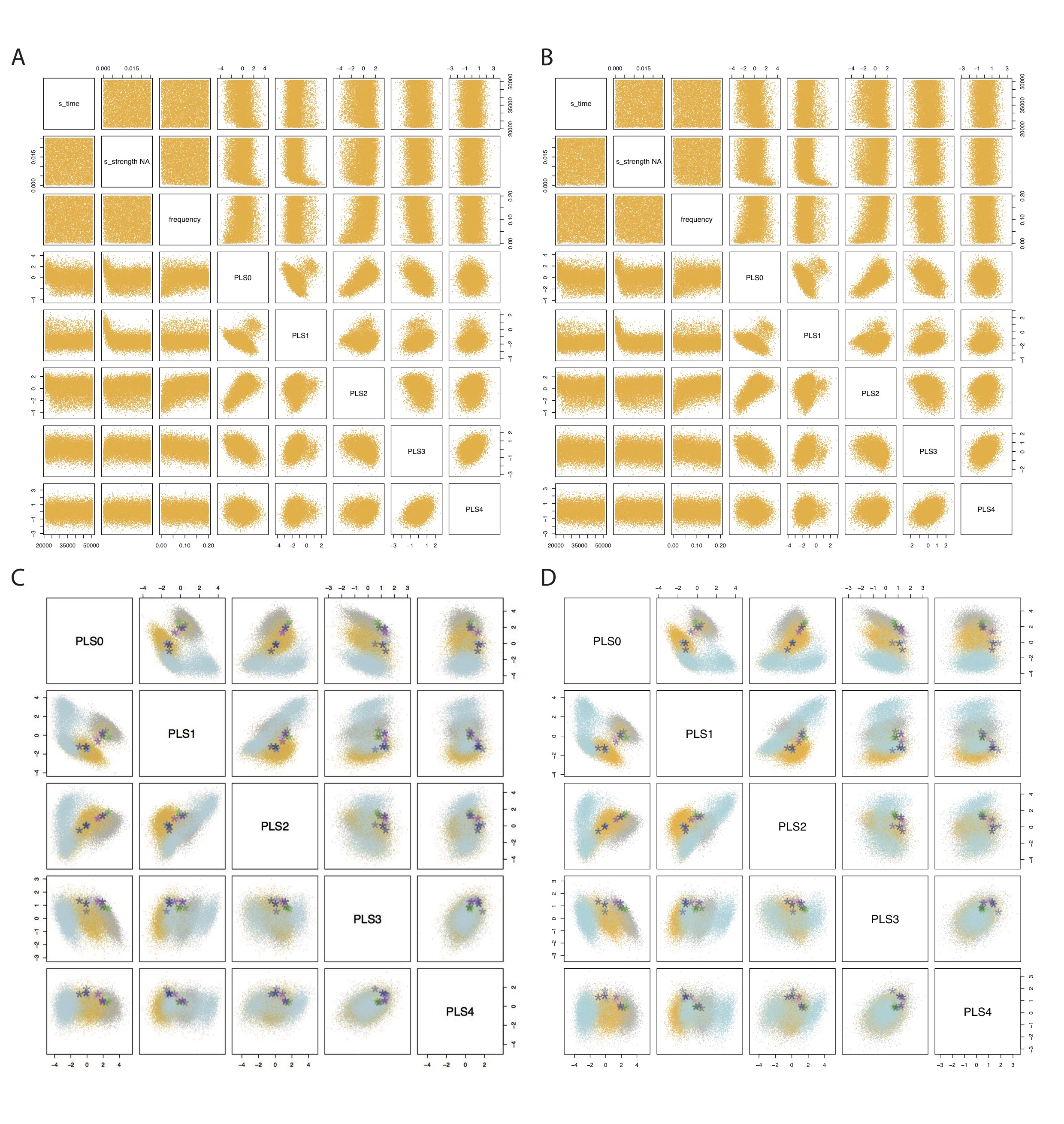

Supplement: S11 Fig — Scatter plots of all five PLS components used in the ABC inference for Europe (A & C) and Asia (B & D). (A & B) PLS transformed statistics for the SSV model and their correlation with the three parameters associated with the SSV model: s_time (time when selection started), s_strength NA (selection strength in non-Africa) and frequency (frequency of the allele at s_time). (C & D) The PLS transformed statistics for all three models (SDN in blue, SSV in orange and NTR in grey) and the PLS transformed observations in all non-African populations (color scheme as in Fig 1). (JPG) [file pgen.1007298.s011.jpg]

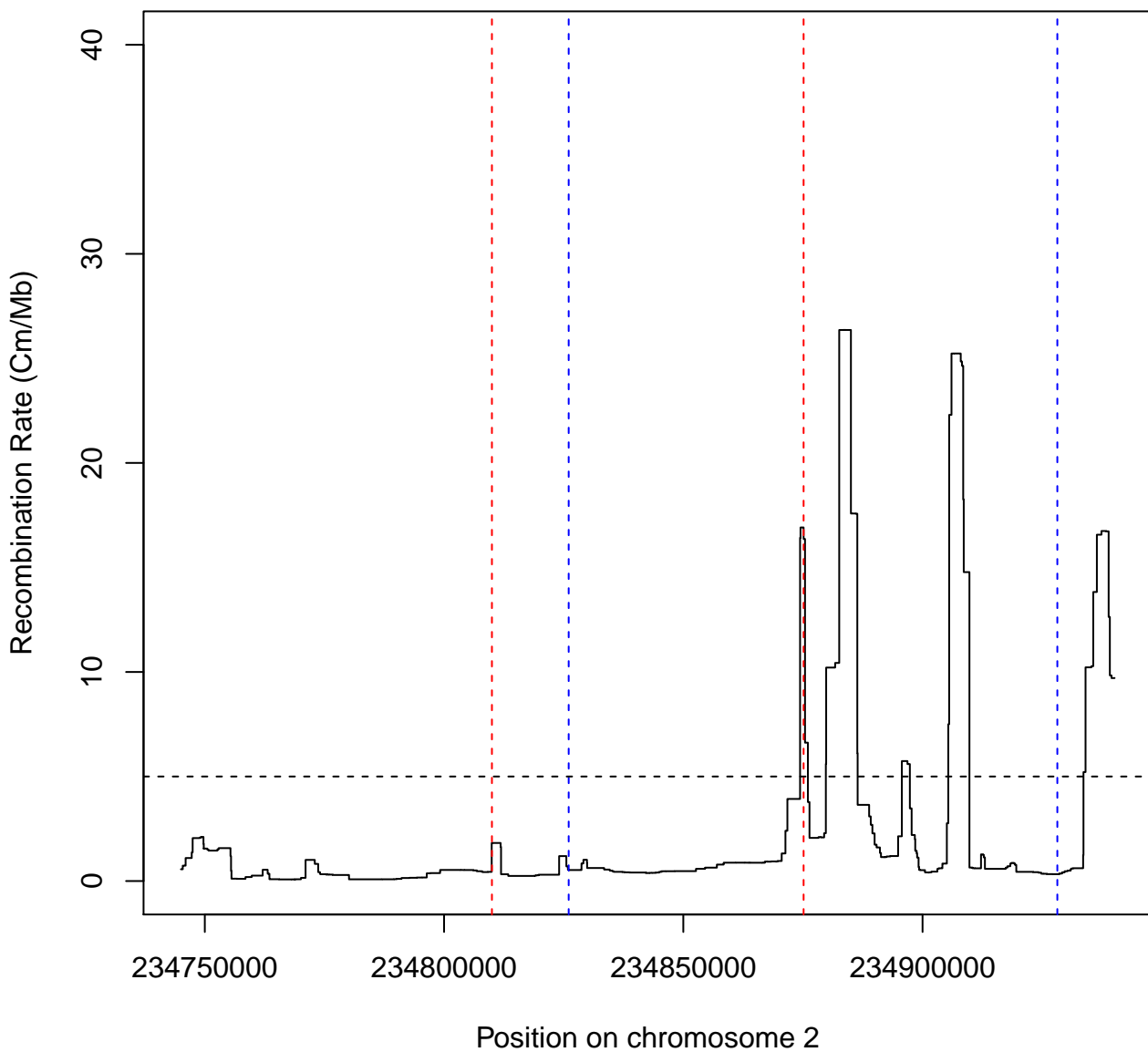

Supplement: S12 Fig — Recombination map based on average recombination rate in two randomly chosen populations per continental group, to avoid biases due to different numbers of populations per continent (YRI, LWK for Africa; GBR, TSI for Europe; CHB, GIH for Asia). The TRPM8 gene is between the two blue vertical dashed lines. The strongly differentiated region is between the two red vertical dashed lines. All basepairs with recombination rates higher than 5 cM/Mb (horizontal dashed line) were considered as being within a hotspot of recombination in the simulations. (PDF) [file pgen.1007298.s012.pdf]

**A**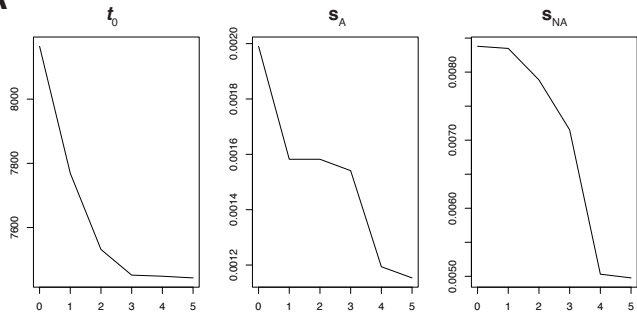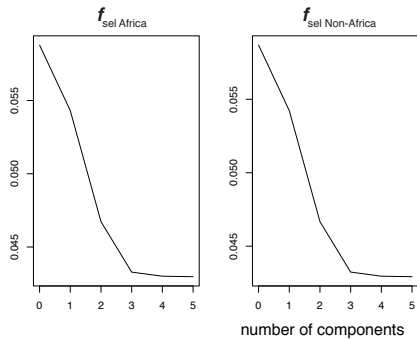**B**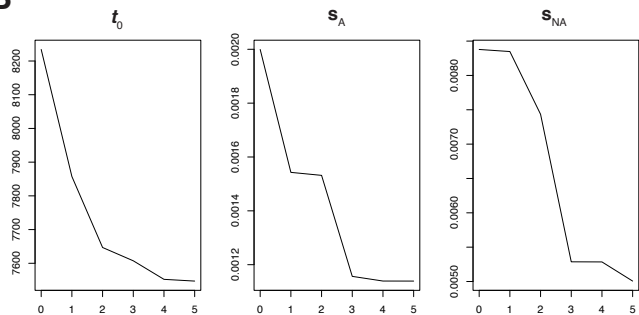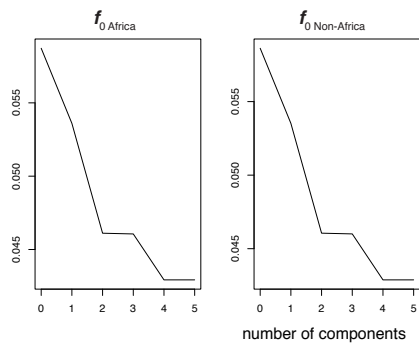

Supplement: S13 Fig — Information contained within each PLS component for a given parameter for all three models combined for (A) the European model and (B) the Asian model. t0 (time when selection started), sA (selection strength in Africa), sNA (selection strength in non-Africa), fsel Africa (frequency of the allele at selection start in Africa), fsel Non-Africa (frequency of the allele at selection start in non-Africa). (PDF) [file pgen.1007298.s013.pdf]
