# Supplementary material for: Human local adaptation of the TRPM8 cold receptor along a latitudinal cline
Source: PLoS Genet. 2018 May 3;14(5):e1007298. doi: 10.1371/journal.pgen.1007298 (PMC5933706; doi:10.1371/journal.pgen.1007298)
Supplement: S1 Table — (A) Diversity estimates measured by means of the number of pairwise differences for all haplotypes carrying the derived rs10166942 T allele. (B) Same as in A after removing haplotypes with evidence of recombination (see Materials and methods and S3 and S4 Figs). (DOCX) [file pgen.1007298.s014.docx]

**A**

| **Population** | **Mean pairwise differences** |
| --- | --- |
| BEB | 4.82 |
| IBS | 5.98 |
| CHS | 6.43 |
| FIN | 6.91 |
| YRI | 7.20 |
| ITU | 7.74 |
| TSI | 7.76 |
| PJL | 8.11 |
| LWK | 8.22 |
| CEU | 9.33 |
| MSL | 9.50 |
| STU | 10.40 |
| GBR | 10.71 |
| GIH | 11.42 |
| CDX | 11.62 |
| CHB | 13.41 |
| ESN | 14.00 |
| KHV | 16.01 |
| JPT | 16.56 |
| GWD | 22.48 |

**B**

| **Population** | **Mean pairwise differences** |
| --- | --- |
| CHS | 3.69 |
| BEB | 4.14 |
| CHB | 4.17 |
| JPT | 4.57 |
| CEU | 4.62 |
| FIN | 4.64 |
| GBR | 4.70 |
| PJL | 4.72 |
| GIH | 4.72 |
| IBS | 4.74 |
| KHV | 4.74 |
| STU | 4.76 |
| ITU | 4.81 |
| TSI | 5.30 |
| CDX | 5.41 |
| YRI | 7.20 |
| GWD | 7.80 |
| LWK | 8.22 |
| MSL | 9.50 |
| ESN | 10.00 |
