# Supplementary material for: Human local adaptation of the TRPM8 cold receptor along a latitudinal cline
Source: PLoS Genet. 2018 May 3;14(5):e1007298. doi: 10.1371/journal.pgen.1007298 (PMC5933706; doi:10.1371/journal.pgen.1007298)
Supplement: S3 Table — Full model stability estimates for each fixed and random effect in each analysis (original estimate obtained from the full data set and the range of estimates derived from omitting individuals and populations (GLMM) or populations (PGLS), one at a time). The small ranges around the original value indicate the overall good stability of the model. Based on z-transformed predictor variables for the PGLS analysis and the GLMM analysis of the SGDP data. (DOCX) [file pgen.1007298.s016.docx]

|  | **Predictor** | **original** | **min** | **max** |
| --- | --- | --- | --- | --- |
| **1KGP**  **PGLS** | (Intercept) | 0.4099 | 0.3607 | 0.4154 |
|  | Annual mean temperature^a^ | 0.0448 | 0.0266 | 0.0797 |
|  | Latitude^a^ | 0.1049 | 0.0990 | 0.1505 |
| **1KGP**  **GLMM** | (Intercept) | -5,038 | -7,896 | -4,596 |
|  | Annual mean temperature | 0.061 | 0.045 | 0.140 |
|  | Latitude | 0.086 | 0.077 | 0.124 |
|  | Distance to YRI | 9,370 | 7,751 | 10,579 |
|  | Individual | 0.096 | 0.000 | 0.453 |
|  | Population | 0.535 | 0.672 | 0.744 |
| **SGDP**  **GLMM** | (Intercept) | 0.172 | 0.124 | 0.203 |
|  | Annual mean temperature^b^ | 0.859 | 0.732 | 1.019 |
|  | Latitude^b^ | 1.379 | 1.312 | 1.574 |
|  | Distance to YRI | 0.661 | 0.598 | 0.752 |
|  | Individual | 0.002 | 0 | 0.07 |
|  | Population | 1.109 | 1.022 | 1.064 |

^a^ Z-transformed to have a mean of 0 and a standard deviation of 1. Degree of Celsius (Temperature) original values had a mean of 20.98 and a standard deviation of 7.44. Degree of latitude original values had a mean of 25.9 and a standard deviation of 17.4.

^b^ Z-transformation to a mean of 0 and a standard deviation of 1. Degree of Celsius (Temperature) original values had a mean of 15.61 and a standard deviation of 10.22. Degree of latitude original values had a mean of 28.01 and a standard deviation of 21.39.
