# Supplementary material for: Human local adaptation of the TRPM8 cold receptor along a latitudinal cline
Source: PLoS Genet. 2018 May 3;14(5):e1007298. doi: 10.1371/journal.pgen.1007298 (PMC5933706; doi:10.1371/journal.pgen.1007298)
Supplement: S4 Table — Power of ABC analysis to correctly assign the model in simulations of European and Asian demography using 10,000 random samplings. TP (True Positive), FP (False Positive), and FN (False Negative). (DOCX) [file pgen.1007298.s017.docx]

|  |  | **TP** | **FP** | **FN** |
| --- | --- | --- | --- | --- |
| **Europe** | NTR | 0.9539 | 0.0278 | 0.0461 |
|  | SDN | 0.9637 | 0.0948 | 0.0363 |
|  | SSV | 0.7987 | 0.01925 | 0.2013 |
| **Asia** | NTR | 0.9528 | 0.0289 | 0.0472 |
|  | SDN | 0.9606 | 0.08475 | 0.0394 |
|  | SSV | 0.8119 | 0.0237 | 0.1881 |
