# Supplementary material for: Human local adaptation of the TRPM8 cold receptor along a latitudinal cline
Source: PLoS Genet. 2018 May 3;14(5):e1007298. doi: 10.1371/journal.pgen.1007298 (PMC5933706; doi:10.1371/journal.pgen.1007298)
Supplement: S5 Table — Bayes factor (measure of confidence) and the resulting posterior probability (Post. Prob.) for the SSV model in each population, ordered by latitude. t0: time when selection starts; SNA: selection strength in non-African population (ceased 3,000 years ago); fsel: frequency of allele at selection start. The median of the posterior distribution of each inferred parameter is shown together with its 95% confidence interval (2.5%–97.5%). (DOCX) [file pgen.1007298.s018.docx]

| Population | Bayes Factor | Post. Prob. | t_0_ (in years)  median 2.5% 97.5% | | | S_NA_ (in %)  median 2.5% 97.5% | | | f_sel_ (in %)  median 2.5% 97.5% | | |
| --- | --- | --- | --- | --- | --- | --- | --- | --- | --- | --- | --- |
| FIN | 19.6 | 0.952 | 33634 | 21806 | 49784 | 1.328 | 0.345 | 2.442 | 0.075 | 0.011 | 0.188 |
| GBR | 12776.7 | 1.000 | 30882 | 21482 | 48821 | 1.411 | 0.357 | 2.432 | 0.071 | 0.011 | 0.184 |
| CEU | 4635.6 | 1.000 | 31362 | 21439 | 49054 | 1.438 | 0.403 | 2.448 | 0.079 | 0.013 | 0.188 |
| TSI | 37.1 | 0.974 | 35507 | 22087 | 49674 | 1.412 | 0.306 | 2.425 | 0.103 | 0.015 | 0.195 |
| IBS | 64.5 | 0.985 | 33197 | 21406 | 49490 | 1.203 | 0.294 | 2.442 | 0.085 | 0.011 | 0.191 |
| CHB | 409.4 | 0.998 | 24031 | 21036 | 47190 | 0.316 | 0.061 | 0.714 | 0.074 | 0.010 | 0.190 |
| JPT | 60.2 | 0.984 | 24816 | 21036 | 47871 | 0.319 | 0.068 | 0.760 | 0.076 | 0.008 | 0.189 |
| PJL | 338.9 | 0.997 | 24577 | 21089 | 48293 | 0.453 | 0.119 | 2.276 | 0.072 | 0.007 | 0.189 |
| BEB | 22.6 | 0.958 | 26402 | 21072 | 48311 | 0.335 | 0.079 | 0.854 | 0.078 | 0.005 | 0.188 |
| GIH | 53.6 | 0.982 | 25647 | 21060 | 48397 | 0.363 | 0.088 | 1.044 | 0.073 | 0.005 | 0.186 |
| CHS | 2079.7 | 1.000 | 23944 | 21028 | 47186 | 0.323 | 0.051 | 0.787 | 0.069 | 0.008 | 0.184 |
| CDX | 7.5 | 0.882 | 25982 | 21121 | 48365 | 0.265 | 0.032 | 0.816 | 0.073 | 0.007 | 0.188 |
| ITU | 20.3 | 0.953 | 25818 | 21064 | 48141 | 0.283 | 0.043 | 0.814 | 0.072 | 0.005 | 0.186 |
| KHV | 4.9 | 0.829 | 26461 | 21078 | 48968 | 0.230 | 0.025 | 0.790 | 0.077 | 0.007 | 0.189 |
| STU | 33.1 | 0.971 | 25549 | 21080 | 48141 | 0.296 | 0.044 | 0.928 | 0.069 | 0.004 | 0.186 |
