# Supplementary material for: Human local adaptation of the TRPM8 cold receptor along a latitudinal cline
Source: PLoS Genet. 2018 May 3;14(5):e1007298. doi: 10.1371/journal.pgen.1007298 (PMC5933706; doi:10.1371/journal.pgen.1007298)
Supplement: S7 Table — Migraine prevalence per country gathered from Stovner et al. [57]. When multiple samplings per population were available, mean migraine prevalence or mean DAF reported. Pearson correlation between DAF and migraine prevalence: rho = 0.61 (p-value = 0.11). (DOCX) [file pgen.1007298.s020.docx]

| Country | 1000 Genomes | DAF rs10166942 | Migraine prevalence (in %) |
| --- | --- | --- | --- |
| Nigeria | YRI/ESN | 0.05 | 6 |
| China | CHB/CHS/CDX | 0.35 | 4.9 |
| Japan | JPT | 0.45 | 7.2 |
| India | GIH/ITU | 0.46 | 11 |
| Spain | IBS | 0.8 | 12 |
| Italy | TSI | 0.84 | 6.2 |
| UK (Eng/Scot) | GBR | 0.8 | 14.3 |
| Finland | FIN | 0.87 | 11.6 |
